# Visual Discomfort and Variations in Chromaticity in Art and Nature

**DOI:** 10.3389/fnins.2021.711064

**Published:** 2021-12-20

**Authors:** Olivier Penacchio, Sarah M. Haigh, Xortia Ross, Rebecca Ferguson, Arnold J. Wilkins

**Affiliations:** ^1^School of Psychology and Neuroscience, University of St. Andrews, St. Andrews, United Kingdom; ^2^Department of Psychology, University of Nevada Reno, Reno, NV, United States; ^3^Center for Integrative Neuroscience, University of Nevada Reno, Reno, NV, United States; ^4^Department of Psychology, University of Essex, Colchester, United Kingdom

**Keywords:** visual discomfort, efficient coding, natural scenes, image statistics, color, chromaticity difference, hypermetabolism

## Abstract

Visual discomfort is related to the statistical regularity of visual images. The contribution of luminance contrast to visual discomfort is well understood and can be framed in terms of a theory of efficient coding of natural stimuli, and linked to metabolic demand. While color is important in our interaction with nature, the effect of color on visual discomfort has received less attention. In this study, we build on the established association between visual discomfort and differences in chromaticity across space. We average the local differences in chromaticity in an image and show that this average is a good predictor of visual discomfort from the image. It accounts for part of the variance left unexplained by variations in luminance. We show that the local chromaticity difference in uncomfortable stimuli is high compared to that typical in natural scenes, except in particular infrequent conditions such as the arrangement of colorful fruits against foliage. Overall, our study discloses a new link between visual ecology and discomfort whereby discomfort arises when adaptive perceptual mechanisms are overstimulated by specific classes of stimuli rarely found in nature.

## Introduction

Viewing certain static patterns can result in visual stress, the collective term for a variety of bodily symptoms and perceptual distortions that include discomfort, malaise and nausea, and perceptual instability, hallucinatory colors and shapes (Wilkins, 1995). The patterns responsible are usually those that in patients with photosensitive epilepsy are capable of inducing seizures (Wilkins et al., 1984; Hermes et al., 2017).

As yet, no general principle explains why some stimuli cause visual stress. However, a strong candidate for such a principle is the theory of efficient coding (Barlow, 1961; Simoncelli, 2003). This theory predicts that sensory systems, and the human visual system in particular, have evolved to provide an efficient representation of the stimuli that most commonly appear in natural environments by exploiting their statistical regularities. An efficient representation maximizes information while limiting metabolism (Olshausen and Field, 2004). The theory of efficient coding has received strong empirical support [e.g., see Machens et al. (2005)]. The association between this theory and visual stress rests on the observation that the patterns that cause visual stress are quite unlike the images that generally occur in nature. Natural scenes, despite their diversity, have particular statistical regularities. For example, the luminance values of nearby locations are highly correlated, and spatial correlation decreases with distance remarkably consistently across different scenes. As a consequence, the luminance of natural scenes has a Fourier amplitude spectrum that decreases with increasing spatial frequency according to the reciprocal of frequency, f, as 1/f^α^ with α between 0.5 and 1.5 (Field, 1987; Tolhurst et al., 1992; Geisler, 2008). A range of theoretical works have suggested that within the early visual system there exists a tight adaptation of the coding mechanisms to the structure of natural scenes (Atick and Redlich, 1992; Field, 1994; Olshausen and Field, 1996a; Simoncelli and Olshausen, 2001). Empirical studies have shown that discrimination performance is optimal when stimuli have a 1/f^α^ spectrum (Knill et al., 1990; Parraga et al., 2000; Geisler et al., 2001). Similarly, images with amplitude spectra that depart from 1/f^α^ are usually uncomfortable to look at (Fernandez and Wilkins, 2008; Juricevic et al., 2010; O’Hare and Hibbard, 2011; Penacchio and Wilkins, 2015; Ogawa and Motoyoshi, 2020). Indeed, a simple measure of the departure from 1/f^α^ can predict more than 25% of the variance in judgments of discomfort (Penacchio and Wilkins, 2015).

The strength of the brain response to uncomfortable visual stimuli also suggests a link between the efficient encoding of natural scenes and visual stress. The receptive fields and lateral connectivity of neurons in the primary visual cortex are such that natural scenes produce a sparse cortical response, thereby minimizing metabolic demand (Olshausen and Field, 1996b,^2004^; Vinje and Gallant, 2000). In computational models of the cortex, uncomfortable images have been shown to give rise to a response that is less sparse than for other images (Hibbard and O’Hare, 2015). Images that are uncomfortable usually evoke a large cortical hemodynamic response, measured using fMRI (Huang et al., 2003, 2011) or near infrared spectroscopy (Haigh et al., 2013a,^2015^; Le et al., 2017), and a large electrical response measured in terms of steady state visual evoked potential (O’Hare, 2016; Haigh et al., 2019; Gentile and Aguirre, 2020; Lindquist et al., 2021) or alpha suppression (Haigh et al., 2018). Taken together, converging evidence suggests that specific deviations from the luminance profile typically found in natural scenes causes visual stress and can be associated with increased cortical activity.

Although color underpins an important part of human visual experience, there have been no studies of the way in which the color in natural scenes is related to discomfort. Yet we know that some combinations of colors cause visual stress while other do not: the discomfort from colored gratings, for example, depends on the difference in chromaticity between the component stripes (Haigh et al., 2013a,^2019^; Lindquist et al., 2021). This is the case only when the difference in color is measured in a perceptual color space and not when measured in terms of cone activation (Haigh et al., 2018). Over a large gamut of chromaticity, increasing chromaticity difference consistently increases discomfort (Haigh et al., 2012, 2013a; Lindquist et al., 2021), and evokes a large hemodynamic (Haigh et al., 2013a,^2015^) and electrophysiological response (Haigh et al., 2015, 2019; Lindquist et al., 2021). The effect is not attributable to any influence of chromatic aberration on accommodation (Haigh et al., 2013b).

Here, we explored whether a measure of chromaticity difference within complex images, as opposed to simple patterns, can explain variance in discomfort over and above that already explained in terms of luminance. Moreover, we asked whether the visual stress caused by color arrangements can be understood from the perspective of the theory of efficient coding of natural stimuli, with uncomfortable stimuli being those that deviate from nature, as is the case in respect of luminance. By comparing chromaticity differences found in uncomfortable stimuli to chromaticity differences in natural scenes, we show that large differences in chromaticity are not typically found in nature, and that deviation from natural limits is associated with discomfort.

## Materials and Methods

### Participants

For Experiment 1, 61 participants with self-reported normal color vision and normal visual acuity (53 female, 8 male; mean age 20.10 years, SD age 2.57) took part and rated for discomfort 50 stimuli (see sections “Procedure” and “Stimuli” below). Two participants showed no variability in their responses, which suggests that they did not appropriately engage with the task. They were therefore removed from the analysis (total *N* = 59, 51 female, 8 male; mean age 20.25 years, SD age 2.69).

For Experiment 2, a replication, 62 participants with self-reported normal color vision and normal visual acuity (49 female, 13 male; mean age 19.29 years, SD age 1.78) took part and rated for discomfort 50 new stimuli (see sections “Procedure” and “Stimuli” below). Four participants showed no variability in their responses and were removed from the analysis (total *N* = 58, 46 female, 12 male; mean age 19.15 years, SD age 1.80). Supplementary Figures 1, 2 shows all the raw data for the observers included in the statistical analysis.

For Experiment 3, 61 participants with self-reported normal color vision and normal visual acuity (44 female, 15 male; mean age 20.6 years, SD age 4.3) took part, one of which showed no variability in the responses and was discarded from the analysis (total *N* = 60, 44 female, 14 male; mean age 20.4 years, SD age 4.1).

None of the participants reported a diagnosed psychiatric or neurological condition, and all verified that they had normal or corrected to normal vision. All participants were recruited from the University of Nevada, Reno, and electronically self-consented into the study. Participants were given course credit for their time and were entered into a raffle to win a $10 Amazon gift card. This protocol was approved by the Institutional Review Board at the University of Nevada, Reno (333057), and was conducted in accordance with the Declaration of Helsinki.

### Procedure

All responses were collected remotely *via* Qualtrics in accordance with COVID-19 protocols. For both experiments, participants viewed each image for an unlimited time and were asked to report their level of comfort (No Discomfort, Some Discomfort, Moderate Discomfort, Uncomfortable, and Very Uncomfortable) for each of the 50 images. Responses were coded 1–5 in increasing discomfort.

### Color Metric

**FIGURE 1:**
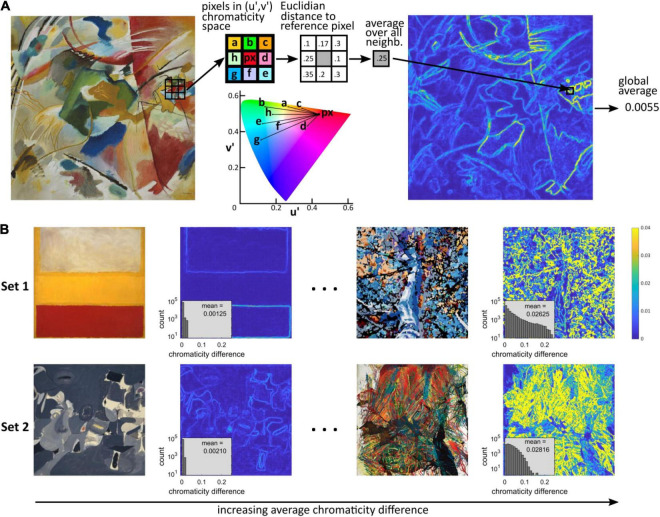
Computation of chromaticity difference. **(A)** Computation of the average chromaticity difference of an image. We computed the u′ and v′ coordinates in the CIELUV chromaticity space for each pixel (px) and its 8-connected neighboring pixels (a to h). We then computed the Euclidian distance between the chromaticity of the reference pixel and the chromaticity of each of the 8 neighboring pixels in the (u′,v′) plane (i.e., independently of any difference in luminance). These distances were then averaged to give the chromaticity difference for each reference pixel, which provided a “heatmap” of local chromatic difference. The color metric, average chromaticity difference, was computed as the average of the local chromaticity differences over the whole image. **(B)** Image with the lowest (left) and highest (right) average chromatic difference in Set 1 (top row) and Set 2 (bottom row), and corresponding heatmap of local chromaticity differences. The plots inserted in the heatmaps show the histograms of local chromatic differences and average value (count shown on a logarithmic scale). The images were cropped from the following works of art: Top left: Wassily Kandinsky, “Painting with green center”; Set 1 left: Mark Rothko, “No. 13”; Right: Randy Honerlah, “Letting Go”; Set 2 left: Arshile Gorky, “Soft Night”; right: Shozo Shimamoto, “Explosion 64-1.”

There is evidence of an association between cortical hypermetabolism and visual discomfort. We therefore wanted to define a metric that modeled cortical activation in response to chromatic stimulation. Given the positive correlation between chromaticity differences and hemodynamic and electrophysiological response in the cortex, we use the local chromaticity differences in an image to construct the metric. Specifically, to compute the color metric (Figure 1A), the original 512 × 512 pixels images in Set 1 and 2 were first down-sampled to 256 × 256 pixels using The Matlab Inc, 2019 function “imresize” with nearest-neighbor interpolation. The down-sampled images were then converted to the CIE XYZ color space and the resulting images converted to the CIE LUV color space using the function “applycform” in the Computational Color Science toolbox (Westland et al., 2012) with respective arguments “makecform(‘srgb2xyz’)” and “makecform(‘xyz2upvpl’).” The third channel (“l”) was then discarded, removing luminance from consideration. Thus, each pixel in the (down-sampled) image corresponded to two chromaticity coordinates (*u*, *v*). We then defined the chromaticity difference of each pair of adjacent pixels *px*_1_ and *px*_2_ to be the Euclidian distance in the (u′,v′) plane, i.e., $d\left(px_{1},px_{2}\right)=\sqrt{{\left(u_{px_{1}}-u_{px_{2}}\right)}^{2}+{\left(v_{px_{1}}-v_{px_{2}}\right)}^{2}}$ (see CIELUV diagram in Figure 1A). The chromaticity difference at a given pixel was then defined as the average chromaticity difference between the pixel and all its adjacent pixels as *d*_*px*_ = (1/*N*)∑_*i*_*d*(*px*, *px*_*i*_), where *i* runs over all the adjacent pixels (*N* = 3 when *px* was in an image corner, *N* = 5 if *px* was on a border but was not a corner, *N* = 8 for all the pixels in a 8-neighborhood otherwise, as represented in Figure 1A, where *px*_*i*_ = *a*, *b*, *c*, *d*, *e*, *f*, *g*, *h*). For each image, the color metric was a single number computed as the average of the pixels’ chromaticity differences over all the pixels in the image (Figure 1B). Note that as an average of distances in the (u′,v′) plane, the color metric can be interpreted as a distance in this space. Our rationale for considering differences in chromaticity and not absolute chromaticity is that while there are effects of absolute chromaticity, as opposed to chromaticity differences, on visual discomfort, differences in chromaticity play a major part in predicting both discomfort from patterns (Haigh et al., 2013a), and the electrical and hemodynamic response of the cortex to a stimulus.

### Stimuli

The stimuli used in Experiment 1 and 2 were obtained by a web search for “Contemporary art.” Only art that did not represent the form of an object was selected. This was to avoid semantic association affecting the ratings of discomfort. We purposely selected art that varied in chromaticity and in structural complexity to ensure enough variability in these two metrics. The images were then resized and cropped to 512 × 512 pixels at 300 dpi. Both sets of stimuli are shown in Supplementary Figures Set 1, 2. In Experiment 3, our aim was to create groups of three stimuli (triples) with the same amount of luminance edge energy but three different levels of average chromaticity difference. To this aim, we first defined three levels for the metric (“low,” “medium,” and “high”) that matched the lower end, the mean and the higher end of the distribution of the images in Set 1. Next, we applied a set of 350 transformations to each image in Set 1 using rotations and pseudo-rotations in the (u′,v′) plane of the CIELUV space and switched back to sRGB images while preserving the luminance content of the images. The full procedure is described in Supplementary Material (page 9; see section “Data Availability Statement” for code). To keep the number of stimuli to rate for discomfort the same as in Experiment 1 and 2, we sampled 25 triples. Accordingly, the stimuli of Experiment 3 consisted of 25 triples in which each triple was made of three transformed versions of the same stimulus in Set 1, with three different levels of average chromaticity difference but the same amount of luminance edge energy.

### Natural Scenes

For comparison purposes, we computed the above color metric for a large set of natural scenes. We considered two databases of calibrated natural images: Geisler and Perry’s database of scenes from nature (Geisler and Perry, 2011) and Párraga’s Barcelona calibrated database (Vazquez-Corral et al., 2009). The average chromaticity difference of all the 2,844 × 4,284 pixels sRGB images in Geisler and Perry’s sets 1, 2 and 3 (*N* = 576) was computed following the steps described above. For the Barcelona calibrated database, we considered all the sets of images that did not include man-made objects (i.e., “Snow and Seaside” and “Naturalistic 1–3,” *N* = 256). To compute the color metric for these sets, we followed the process described above except that we started with the calibrated 756 × 1,134 pixels images in CIE XYZ space, as the provided RGB images are not calibrated, and first cropped the images to 756 × 756 pixels by eliminating the first left third containing the calibration gray ball. Overall, the process provided two estimates for the distribution of the average chromaticity difference for natural images.

To understand better the range of variations of the color metric in natural scenes, we withdrew 25 patches of size 256 × 256 pixels at random locations using a uniform distribution in all the images in sets “Naturalistic 2 and 3” of the Barcelona calibrated database, resulting in 1,750 patches for each set. We then computed their average chromaticity difference. These two sets were chosen because they contain a large sample of the features of natural scenes, from chromatically uniform green foliage or brown ground, contrast between green foliage and blue sky, and a range of colorful fruits of different sizes, framed at different distances.

### Statistical Analysis

In Experiments 1 and 2, we wanted to estimate the effect of average chromaticity difference and departure with respect to 1/f^α^ in the general population while ignoring the particularities of the responses by a participant, which tend to be correlated across images. Correlations for each participant are particularly expected for online experiments, as opposed to lab experiments, because the experimental conditions (viewing conditions, display device) are different for each observer. To analyze the effect of all these factors we used linear mixed effects (multilevel) models. We fitted the models using the function “lmer” in the package lme4 (Bates et al., 2015) in R (R Core Team, 2020). Average chromaticity difference and departure from 1/f^α^ were considered as fixed effects, and observer as a random effect, here also considered to include the experimental setting and conditions, which differed between observers. To estimate the effect of average chromaticity difference in Experiment 3 while ignoring the different amounts of luminance edge contrast between triples, we considered the level of the metric (“low,” “medium,” and “high”) as a fixed effect, and observer and triple identity as random effect. Multiple comparisons between levels of the predictor were done using the Tukey procedure from the R package multcomp (Hothorn et al., 2008). We used information criteria (AIC, BIC) and log likelihood for model selection and used likelihood ratio for hypothesis testing (comparing with χ^2^-distributions with a degree of freedom given by the difference of degrees of freedom of the compared models). Following recommended practice (Meteyard and Davies, 2020), we detail all the mixed effects models tested and the final models adopted (see Supplementary Results, “Statistical inference”). Previous studies on visual discomfort reported Spearman’s ρ correlations between image metric and ratings for discomfort averaged across observers. To allow for comparison, we also report these statistics, as well as their robustness with 95% confidence intervals computed using a standard bootstrap procedure (Rousselet et al., 2019). Test of significance between Spearman correlations were done using the same method. All bootstraps were computed with 10,000 replicates.

## Results

### Experiments 1 and 2: Self-Reported Visual Discomfort and Average Chromaticity Difference

**FIGURE 2:**
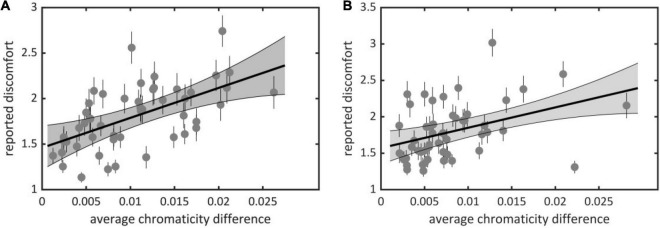
Average reported discomfort against average chromaticity difference for **(A)** Experiment 1 (Set 1) and **(B)** Experiment 2 (Set 2). Each point represents an image in one of the sets with (x-coordinate) its average chromaticity difference and (y-coordinate) reported visual discomfort averaged over all observers. Plain lines represent best linear fit, the gray area represents the 95% confidence interval for the regression line and the small lines represent the standard error of the mean (see Supplementary Material for individual data).

We found a significant effect of average chromaticity difference on observers’ judgments of discomfort for both experiments (Set 1, χ^2^ = 215.72, df = 3, *p* < 10^–15^; Set 2, χ^2^ = 102.68, df = 3, *p* < 10^–15^); visual discomfort increased with average chromaticity difference [Set 1, Figure 2A, slope estimate 0.34, 95% ci = (0.25, 0.44); Set 2, Figure 2B, 0.22, ci = (0.15, 0.30)]. Although robust, the effect of averaged chromaticity difference on discomfort varied considerably between observers, as can be seen from the best linear fit for individual raw data (see Supplementary Figures 1, 2). Minimum, maximum, and standard deviation of the distribution of individual slopes over observers were, respectively, 0.016, 1.13, and 0.26 in Set 1 and −0.13, 0.48, and 0.17 in Set 2. The Spearman’s ρ correlation between mean ratings and the color metric was 0.62 [ci = (0.42, 0.76)], for Set 1 and 0.43, ci = (0.15, 0.67), for Set 2.

**FIGURE 3:**
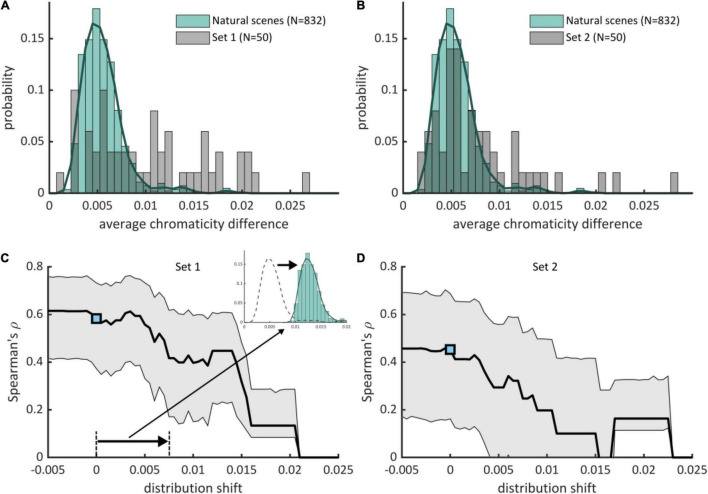
Positive deviation of average chromaticity difference with respect to natural scenes drives visual discomfort. **(A,B)** (pale green boxes) Histograms and (green curve) estimated distributions of average chromaticity difference in natural scenes (*N* = 832) with superimposed distribution of average chromaticity difference for the stimuli used in **(A)** Experiment 1 (Set 1, 50 stimuli) and **(B)** Experiment 2 (Set 2, 50 stimuli). Part of the stimuli have a high average chromaticity difference with respect to typical natural scenes. **(C,D)** (black curve) Spearman’s ρ correlations between reported visual discomfort and the rectified z-scores of the average chromaticity difference with respect to the distribution for natural scenes when the distribution is shifted toward lower or higher values of chromaticity differences for **(C)** Set 1 and **(D)** Set 2. The reference distribution for natural scenes corresponds to shift = 0, with the associated correlation shown by a blue point [**(C)**, Experiment 1, 0.62, ci = (0.42, 0.76); **(D)**, Experiment 2, 0.43, ci = (0.15, 0.67)]. Gray-shaded intervals correspond to 95% confidence intervals for Spearman’s ρ correlations. Correlations decrease when the distribution for natural scenes is shifted toward positive values, showing that positive deviation of average chromaticity difference with respect to the reference distribution for natural scenes (shift = 0) is the best predictor of visual discomfort.

We also contrasted discomfort judgments with a previous metric based on luminance only that measures to what extent the two-dimensional amplitude spectrum of an image departs from the 1/f^α^ average amplitude spectrum of natural scenes (Penacchio and Wilkins, 2015). There was a significant effect of departure from 1/f^α^ on discomfort for both sets (Set 1, χ^2^ = 81.50, df = 3, *p* < 10^–15^; Set 2, χ^2^ = 136.58, df = 3, *p* < 10^–15^). The Spearman correlation between mean ratings and departure from 1/f^α^ was 0.51 [ci = (0.27, 0.69)] in Set 1 and 0.22 [ci = (−0.10, 0.51)] in Set 2. We found a correlation between the two metrics [Set 1, Spearman correlation *r* = 0.75, ci = (0.59, 0.85); Set 2, *r* = 0.66, ci = (0.45, 0.81)]. This correlation is expected from the partial correlation between luminance and color edges found in natural scenes (Hansen and Gegenfurtner, 2009). Nonetheless, a model including both metrics improved significantly over a model including average chromaticity difference for Set 2 (χ^2^ = 51.88, df = 1, *p* < 10^–12^, ΔAIC = 7,954 – 7,904 = 50) or departure from 1/f^α^ only (χ^2^ = 35.57, df = 3, *p* < 10^–8^, ΔAIC = 7,934 – 7,904 = 30), suggesting that both color and luminance influenced the discomfort experienced by observers in Experiment 2. These comparisons also show that the relative contribution of average chromaticity difference was stronger in Set 1 than in Set 2. This is likely to reflect the higher spread and average of the distributions of chromaticity difference in Set 1 than Set 2 (mean in Set 1 0.0105 vs. 0.0079 in Set 2, Kolmogorov-Smirnov *D* = 0.28, *p* = 0.0317; median, 0.0098, resp. 0.0063; standard deviation 0.0061, resp. 0.0053, Figures 3A,B).

### Experiment 3: Controlling for Luminance Edge Energy

**FIGURE 4:**
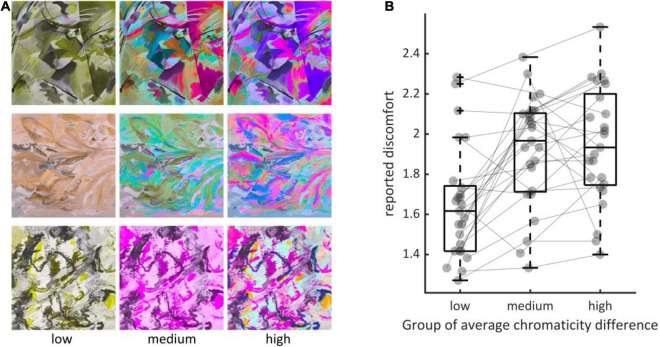
**(A)** Examples of triples of stimuli (rows) showing the three levels of the metric (left column, “low,” middle, “medium” and right, “high”). **(B)** Distributions of average reported discomfort for the three groups defined by the three levels of average chromaticity difference, namely “low” (*N* = 25, mean = 0.0035, std = 0.0009), “medium” (*N* = 25, 0.0115, 0.0009) and “high” (*N* = 25, 0.0190, 0.0018). Every dot represents the reported discomfort averaged over all observers in Experiment 3 when viewing an image with one of the three possible levels of the metric within each triple. The three elements making a triple are joined using the thin gray line. Jitter along the x-axis within each group has been added for visualization purpose. Boxplots display the median, the 25th and 75th percentiles (lower and upper hinges), the lowest measured values within Q1 (first quantile) and 1.5 × Q1 (lower whisker) and the highest observed value within Q3 (third quantile) and 1.5 × Q3 (upper whisker).

To distinguish between the effect of luminance edges from the effect of chromaticity difference on visual discomfort, we compared visual discomfort between levels of average chromaticity difference within triples of stimuli made of an image with a low level, an image with a medium and an image with a high level for the metric but the same level of luminance contrast (Experiment 3). We found a significant effect of the level of average chromaticity difference within triples (χ^2^ = 614.96, df = 13, *p* < 10^–15^, ΔAIC = 11,670 – 11,110 = 560, Figure 4), with considerable variations between observers, as in Experiments 1 and 2 (see Supplementary Figure 3). Visual discomfort was 1.64 ci = (1.454, 1.850) for the low level of the metric, 0.268 ci = (0.124, 0.413) for the difference between the medium and low levels, and 0.294 ci = (0.129, 0.461) for the difference between the low and the high level, with a significant difference between “low” and “medium,” “low and high,” but no significant difference between “medium” and “high” (Tukey adjusted difference between levels: “medium–low,” *z* = 8.58, *p* < 10^–4^; *z* = 9.41, *p* < 10^–4^; *z* = 0.27, *p* = 0.687).

### Comparison With Statistics of Average Chromaticity Difference in Natural Scenes

The distributions of average chromaticity difference of the two databases of natural scenes were similar (Supplementary Figure 4). We therefore joined them to form a single distribution hereafter referred to as the distribution for natural scenes (green curve, Figures 3A,B). Average chromaticity differences for natural scenes ranged between 0.0021 and 0.0198, with 97% of the distribution below 0.010. Mean (0.0056), median (0.0052) and standard deviation (0.0023) of the distribution were lower than those of the two sets of abstract art. We quantified how average chromaticity difference of the stimuli in the two experimental sets deviated from the values typically found in natural scenes. We computed the z-score of the color metric for the two sets of images of abstract art with respect of the distribution for natural scenes. We only considered “positive” deviations with respect to natural scenes by rectifying the z-scores, i.e., sending negative z-scores to zero. Our rationale was that the color arrangements of stimuli with a low average chromaticity difference, which have a “negative deviation” with respect to natural scenes, are not expected to cause discomfort. For both sets of stimuli, the Spearman correlation obtained with the rectified deviations was not significantly lower than those obtained with the original values (Set 1, Spearman’s ρ 0.62 for the reference metric vs. 0.59 for the rectified z-scores, *p* = 0.41; Set 2, 0.43 for both, *p* = 0.50). This shows that the positive association between average chromaticity difference and reported discomfort is conserved even if we consider only positive deviation with respect to natural scenes: visual discomfort increases as the chromaticity differences exceed those from natural scenes.

We further tested the association between experienced discomfort and positive deviation in chromaticity with respect to natural scenes. To this end, we simulated several values of shift of the distribution for natural scenes toward lower and higher values of average chromaticity difference. For each shift, we computed the resulting rectified z-scores of the stimuli in the two sets of abstract art and assessed how they correlated with reported discomfort (Figures 3C,D). We found that correlations decreased with increasing positive shift of the reference distribution, showing that deviation with respect to the real distribution has a better predictive power.

**FIGURE 5:**
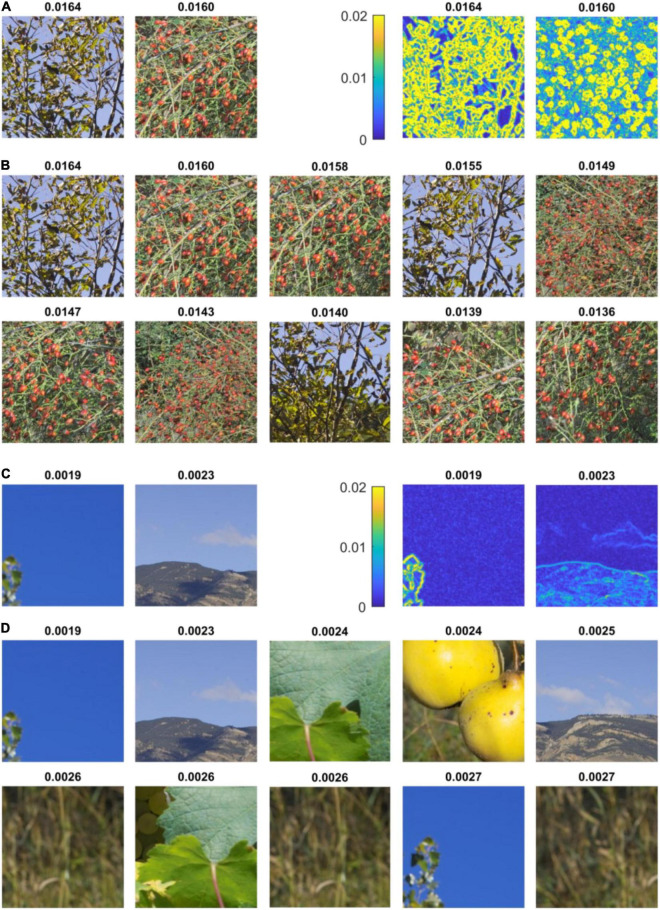
Highest and lowest values of average chromaticity difference in natural scenes. **(A)** (left) First and second random patches with highest average chromaticity difference in set “Naturalistic 3” of the Barcelona calibrated database (Vazquez-Corral et al., 2009) and (right) respective heat maps of local chromaticity difference. The Average chromaticity difference in shown at the top of each inset. The first patch represents tree leaves (a species of genus ash, *Fraxinus*) seen against a blue sky. The second patch shows ripe fruits of dog rose (*Rosa canina*) against green foliage. The heat maps show that chromaticity difference is high at the outline of leaves and fruits. **(B)** Ten first patches with highest average chromaticity difference in the same set, showing leaves against the sky and ripe fruits. **(C)** (left) Last two random patches of lowest average chromaticity difference in set “Naturalistic 3” of the Barcelona calibrated database and (right) respective heat maps of local chromaticity difference. The first patch shows a uniform blue sky and the second patch a mountain landscape. **(D)** Ten last patches with lowest average chromaticity difference in the same set of natural scenes. The patches show a uniform sky, mountain landscapes, close-up of leaves of vineyard and dried wild grasses. See Supplementary Figures 5–16 for a more comprehensive view of the extremes values of average chromaticity differences found in natural scenes.

Can the high values of average chromaticity difference found in some of the stimuli used in Experiments 1 and 2 be reached in natural scenes? To test the extent of local variations of chromatic difference in nature, we considered many small patches in two subsets of the Barcelona calibrated database (“Naturalistic 2–3”) and computed their average chromaticity difference. These two sets were considered because they both contain scenes with both a narrow and a wide range of chromaticities. Importantly, they show a range of scenes with red ripe fruits against a green foliage, a class of stimuli thought to have played a central role in the evolution of trichromacy in primates (Osorio and Vorobyev, 1996; Sumner and Mollon, 2000). We found that the patches at the top of the distribution, i.e., with the highest value of average chromaticity, showed green leaves against the sky, and, mainly, different varieties of ripe fruits against a foliage (Figures 5A,B and Supplementary Figures 5–10). For these stimuli, made of scattered items against a chromatically contrasting background, chromaticity difference is high at the outline of the items (see heat maps in Figure 5A), which results in high values of local average chromaticity difference. By contrast, the lower end of the distribution consists of parts of natural scenes with reduced color contrast (Figures 5C,D and Supplementary Figures 11–16). Taken together, we found that chromaticity difference was higher in the visually uncomfortable stimuli than in natural scenes, with only a specific class of natural stimuli, essentially formed of arrangement of ripe fruits against foliage, approaching (but not exceeding) the values of the color metric associated with the highest discomfort.

## Discussion

We focused on the contribution of chromaticity to visual discomfort to complement previous studies of the effects of luminance contrast. In an initial study with replication (Experiments 1 and 2), we found that averaging local separations in chromaticity (a simple metric with no parameters that simulates cortical activation in response to color contrast) provides a good predictor of visual discomfort. In the online experiments it was not possible to use calibrated displays or to identify any participants with color vision anomalies using specialized equipment, but it is reasonable to suppose that both restrictions would have increased the experimental noise. The predictions were strong, nevertheless. The metric accounts for variance in judgments of discomfort over and above that previously explained (Penacchio and Wilkins, 2015) on the basis of the luminance content of the images. To confirm the influence of average chromaticity difference on visual discomfort independently of the influence of luminance edge contrast, we defined another experiment (Experiment 3) in which we varied chromaticity difference while keeping luminance energy constant. We found the same association between increased chromaticity difference and increased visual discomfort. Finally, we also showed that large chromaticity differences are relatively unusual in the natural environment, except when identifying brightly colored fruit among foliage. This suggests that large chromaticity differences in the natural environment occur but rarely.

The algorithms that predict visual discomfort based on luminance (Penacchio and Wilkins, 2015) support the idea of a link between visual stress and the theory of efficient coding of natural stimuli (Barlow, 1961; Simoncelli, 2003). Discomfort arises when the luminance content of a stimulus deviates consistently from that expected in natural scenes. Uncomfortable stimuli also evoke large metabolic and electrophysiological responses (Huang et al., 2003, 2011; O’Hare, 2016; Le et al., 2017; Haigh et al., 2018, 2019; Gentile and Aguirre, 2020; Lindquist et al., 2021). The discomfort is theorized to serve as a homeostatic signal to avoid stimuli that are computationally and therefore metabolically demanding (Wilkins and Hibbard, 2014). Our findings show that the putative link between visual discomfort and efficient coding extends to color. We estimated the distribution of average chromaticity differences in natural images and showed that positive deviation with respect to this distribution predicted visual discomfort. So once again the deviation from the statistics of natural scenes was associated with discomfort. The cortical representation of color has been shown to be topographically represented as in the perceptual color maps (CIE LUV) in human (Brouwer and Heeger, 2009, 2013) and in monkey brains (Xiao et al., 2003). Stimuli with large average chromaticity differences are therefore associated with strong activation of spatially disparate parts of the cortex. Why do such an activation result in visual discomfort? The underlying mechanism is still to be determined, but, as for the luminance stimuli such as stripes that cause strong gamma oscillations, the spatially scattered stimulation of inhibitory interneurons is likely to play a central role (Honey and Valiante, 2017). The discomfort would, again, be consistent with a homeostatic response that reduces any over-activation resulting from an inefficient encoding of the scene.

Surprisingly, the atypical variations in chromaticity arise from one class of objects: namely arrangements of fruit on trees, at least in our sample of images. The fruit are conspicuous objects, readily identifiable by their chromaticity difference (Osorio and Vorobyev, 1996; Sumner and Mollon, 2000). Although the chromaticity difference was greater for fruit on trees than for more typical natural images, the chromaticity difference for our uncomfortable unnatural stimuli was substantially greater, consistent with an overstimulation of a detection mechanism. The role of chromaticity differences in attracting an observer’s attention has yet to be fully identified but could be a key component in identifying why large color separations are uncomfortable. In the modern environment, where food foraging is a less common activity, but cluttered arrangements of artificial objects with disparate and saturated color are commonplace, sensitivity to large color differences could now have a maladaptive effect giving rise to an atypically strong physiological response that evokes discomfort, and even headaches and seizures in clinical populations.

Linking the overexploitation of an adaptive perceptual process to visual discomfort opens new perspectives on our understanding of visual discomfort. Whilst there is a clear association between some deviations from nature and visual discomfort (Fernandez and Wilkins, 2008; Juricevic et al., 2010; O’Hare and Hibbard, 2011; Penacchio and Wilkins, 2015; Ogawa and Motoyoshi, 2020), not all deviations elicit visual discomfort. For example, square or sine gratings with a low spatial frequency, or images that deviate from 1/f^α^ because they have a *deficit* of contrast energy at mid spatial frequencies, are two classes of stimuli that are rarely found in natural scenes, but are not reported as uncomfortable to look at. By contrast, square and sine gratings with a spatial frequency around 3 cycles per degree, i.e., whose frequency coincides with the maximum of sensitivity of the human visual system (Campbell and Robson, 1968), are strongly associated with visual discomfort and will evoke seizures in some patients with photosensitive epilepsy (Fernandez and Wilkins, 2008; O’Hare and Hibbard, 2011; Penacchio and Wilkins, 2015; Hermes et al., 2017). Deviation from nature is therefore necessary but not sufficient to provoke discomfort. Future work might consider whether the deviations from nature that cause visual discomfort are strong stimuli with chromatic, spatial and/or temporal features that maximally facilitate detection, or, more generally, perceptual mechanisms that are otherwise adaptive in natural environments.

In summary, chromatic contrast plays an important role in determining visual discomfort, a role broadly similar to the role of luminance contrast. Chromaticity difference is a simple metric that can account for substantial variance in judgments of discomfort from images. This metric sets another link between visual stress and the theory of efficient coding of natural stimuli. In nature, chromaticity differences tend to be small, and instances of high chromaticity difference tend to be uncommon and possibly related to foraging. Uncomfortable arrangements of color are not simply different, but exaggerated versions of the chromaticity differences found in nature, and likely to strongly stimulate the visual system. Questions remain as to whether it is generally the case that visual discomfort arises when adaptive perceptual mechanisms are overstimulated by specific classes of stimuli rarely found in nature.

## Data Availability Statement

The datasets presented in this study can be found in online repositories. The color stimuli can be found at https://github.com/SarahMHaigh/DiscomfortComplexImages. The raw individual data, Matlab, and R analysis code are archived at the Dryad digital repository, doi: 10.5061/dryad.bcc2fqzc5.

## Ethics Statement

The studies involving human participants were reviewed and approved by Institutional Review Board at the University of Nevada, Reno (333057). The patients/participants provided their written informed consent to participate in this study.

## Author Contributions

OP, SH, and AW conceived the experiment, analyzed the data, and wrote the manuscript. SH supervised the data collection. XR, RF, and SH collected the data. OP wrote the software for computing chromaticity differences and for formal analysis. All authors contributed to the final version of the manuscript.

## Conflict of Interest

The authors declare that the research was conducted in the absence of any commercial or financial relationships that could be construed as a potential conflict of interest.

## Publisher’s Note

All claims expressed in this article are solely those of the authors and do not necessarily represent those of their affiliated organizations, or those of the publisher, the editors and the reviewers. Any product that may be evaluated in this article, or claim that may be made by its manufacturer, is not guaranteed or endorsed by the publisher.
