# Supplementary material for: Visual Discomfort and Variations in Chromaticity in Art and Nature
Source: Front Neurosci. 2021 Dec 20;15:711064. doi: 10.3389/fnins.2021.711064 (PMC8720932; doi:10.3389/fnins.2021.711064)
Supplement: Supplementary file 1 [file Table_1.pdf]

Electronic Supplementary material

Visual Discomfort and Variations in Chromaticity in Art and Nature

Olivier Penacchio, Sarah M. Haigh, Xortia Ross, Rebecca Ferguson and Arnold J. Wilkins

Supplementary results

Statistical inference

Experiment 1

Models used in the inference process for Experiment 1 (image Set 1). The model chosen is highlighted in grey.

Model	Nested Model	Effects		AIC	BIC	Log Likelihood	Likelihood Ratio test		
		Fixed	Random observer ID (experimental setting)				df	χ^2	p-value
**Mnull**			Intercept	8058	8076	4026			
**MCa**	Mnull	+ colour		7924	7948	3958	1	136.39	$< 10^{-15}$
**MCb**	MCa		+ colour	7848	7884	3918	2	79.34	$< 10^{-15}$
**MCRa**	MCb	+ residual		7857	7899	3922	1	6.87	0.0088
**MCRb**	MCb	+ residual	+ residual	7841	7889	3912	1	11.40	0.0033

Model selected MCb: rating ~ colour + (colour|observer ID)

**Fixed effects**				
	**Estimate**	**SE**	**t-value**	**p-value**
**Intercept**	1.46	0.068	21.58	$< 10^{-15}$
**Colour**	0.34	0.048	7.09	$< 10^{-8}$

**Random effects**		
	**Variance**	**SD**
**Subject**	0.20	0.45
**Colour**	0.090	0.93

Model fit: R^2 (marginal) 0.031; R^2 (conditional) 0.392

Experiment 2

Models used in the inference process for Experiment 2 (image Set 2). The model chosen is highlighted in grey.

Model	Nested Model	Effects		AIC	BIC	Log Likelihood	Likelihood Ratio test		
		Fixed	Random Observer ID (experimental setting)				df	χ^2	p-value
**Mnull**			Intercept	8046	8064	4020			
**MCa**	Mnull	+ colour		7982	8006	3987	1	66.08	$< 10^{-15}$
**MCb**	MCa		+ colour	7954	7990	3971	2	31.76	$< 10^{-6}$
**MCRa**	MCb	+ residual		7904	7946	3945	1	51.88	$< 10^{-12}$
**MCRb**	MCRa		+ residual	7902	7949	3943	1	4.04	0.045

Model selected MCRa: rating ~ colour + residual + (colour|observer ID)

Fixed effects				
	**Estimate**	**SE**	**t-value**	**p-value**
**Intercept**	1.52	0.083	18.71	$< 10^{-15}$
**Colour**	0.086	0.042	2.04	0.0444
**Residual**	0.191	0.025	7.62	$< 10^{-13}$

Random effects		
	**Variance**	**SD**
**Subject**	0.33	0.57
**Colour**	0.048	0.22

Model fit: R^2 (marginal) 0.030; R^2 (conditional) 0.367

Experiment 3

Models used in the inference process for Experiment 3 (triples of stimuli). The model chosen is highlighted in grey.

**Model**	**Nested Model**	**Effects**		**AIC**	**BIC**	**Log Likelihood**	**Likelihood Ratio test**		
		Fixed	Random observer ID (experimental setting)				df	χ^2	p-value
**Mnull**			Intercept	11700	11719	5847			
**MTa**	Mnull	+ metric level (1,2 or 3)		11607	11639	5798	2	96.82	$< 10^{-15}$
**MTb**	MTa		+ random slope for observer ID	11434	11498	5707	5	182.38	$< 10^{-15}$
**MTc**	MTb		+ triple ID as random	11162	11233	5570	1	273.97	$< 10^{-15}$
**MTd**	MTc		+ random slope for triple ID	11110	11213	5539	5	61.79	$< 10^{-11}$

Model selected MTd: rating ~ level + (level|observer ID) + (level|triple ID)

Fixed effects				
	**Estimate**	**SE**	**t-value**	**p-value**
**Intercept (level = 'low')**	1.652	0.101	16.44	$< 10^{-15}$
**level = 'medium'**	0.269	0.073	3.67	6.78×10^{-4}
**level = 'high'**	0.295	0.083	3.50	8.28×10^{-4}

Random effects			
		**Variance**	**SD**
**Observer ID**	level = 'low'	0.42	0.64
	level = 'medium'	0.45	0.67
	level = 'high'	0.48	0.69
**Triple ID**	level = 'low'	0.069	0.26
	level = 'medium'	0.065	0.26
	level = 'high'	0.074	0.27

Model fit: R^2 (marginal) 0.015; R^2 (conditional) 0.466

Supplementary figures

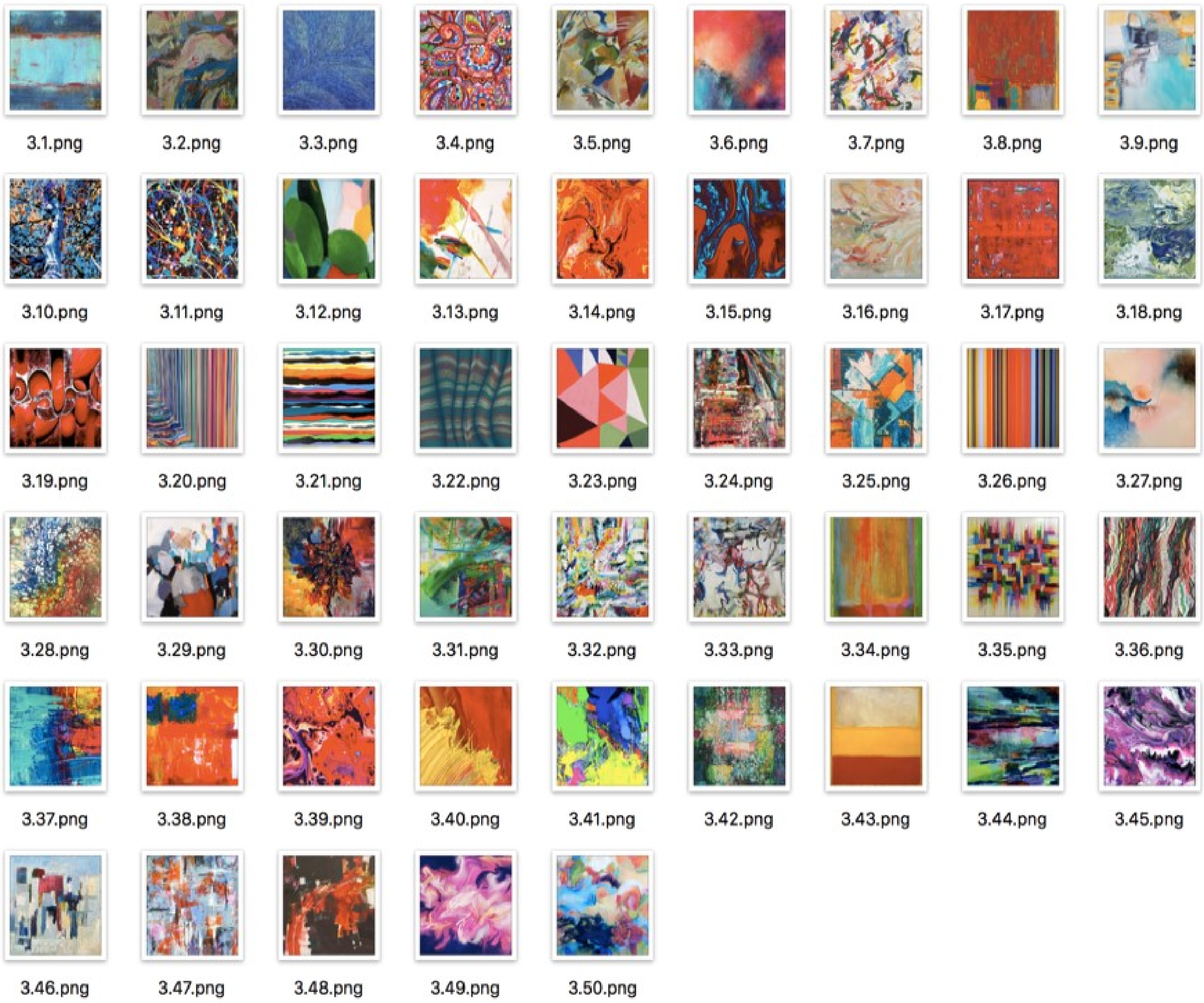

Figure Set 1. Abstract paintings used in Experiment 1.

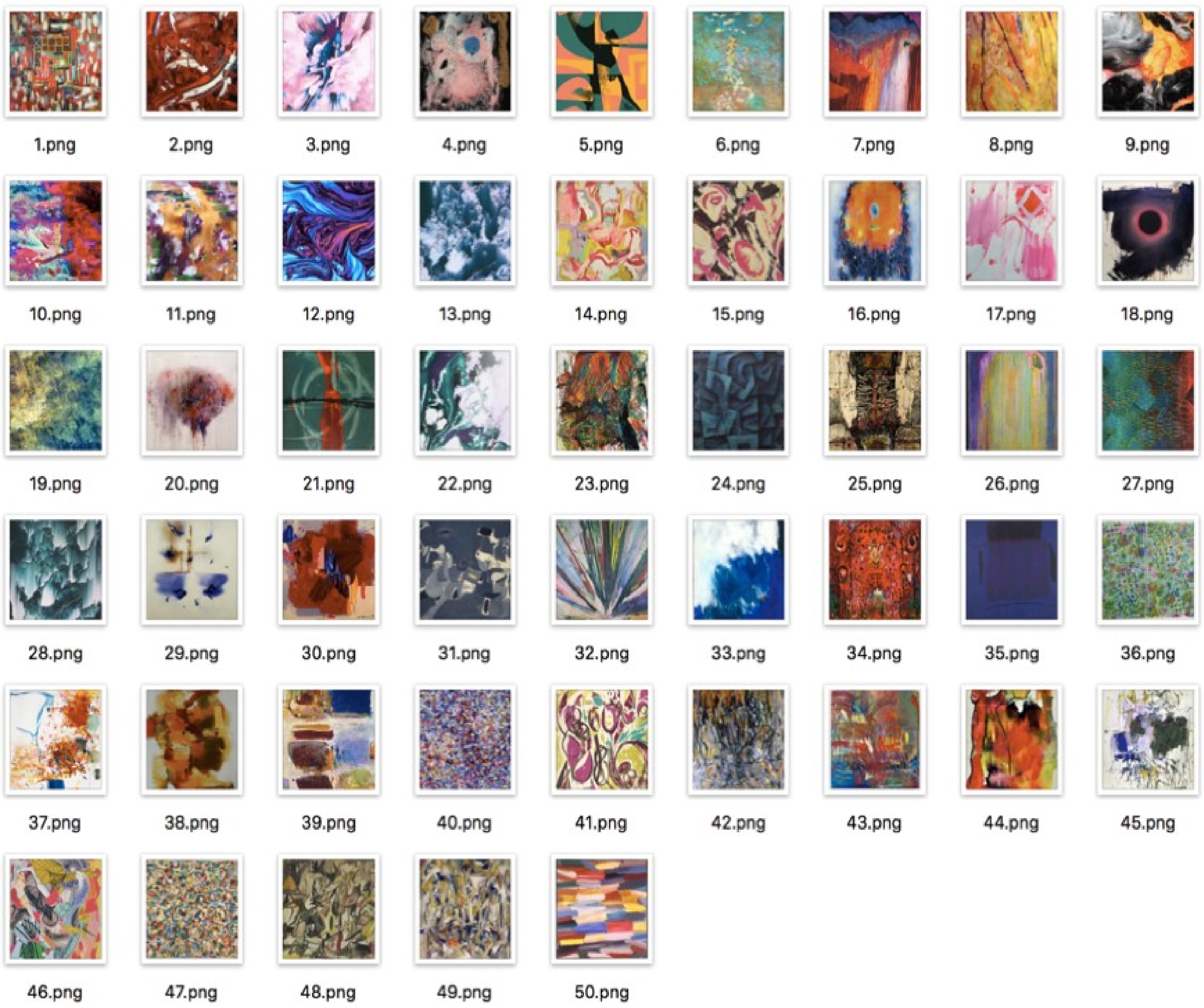

Figure Set 2. Abstract paintings used in Experiment 2.

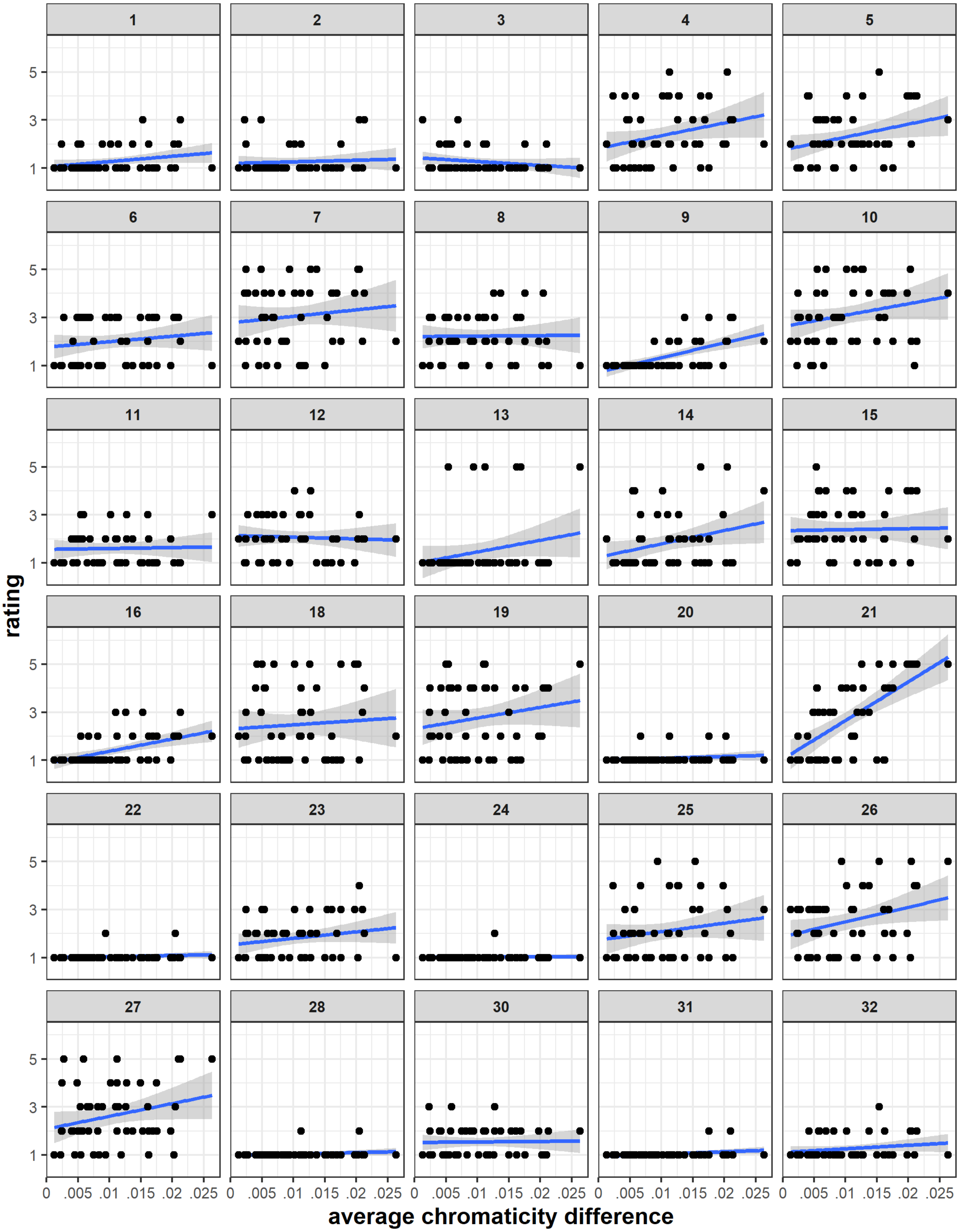

Figure S1, part1. Raw data for all the subjects included in the analysis of Experiment 1 (N = 59). Panel numbers indicate subject identity. Each panel shows the subject's ratings (between 1 and 5, in increasing discomfort) against the images (N = 50) average chromaticity difference. Blue line show best linear fit and shaded area shows 95% confidence intervals. (Figure continues in part 2.)

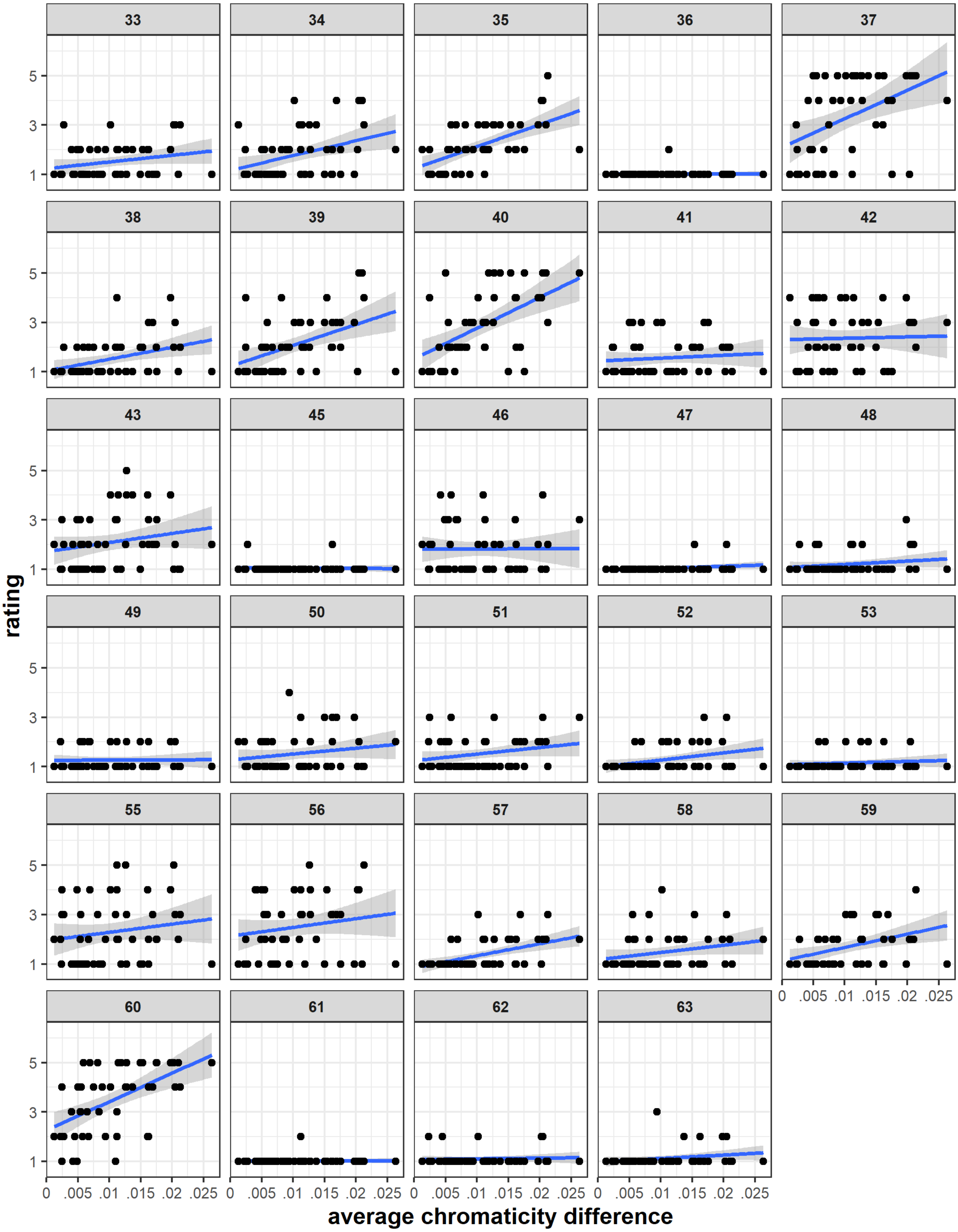

Figure S1, part 2.

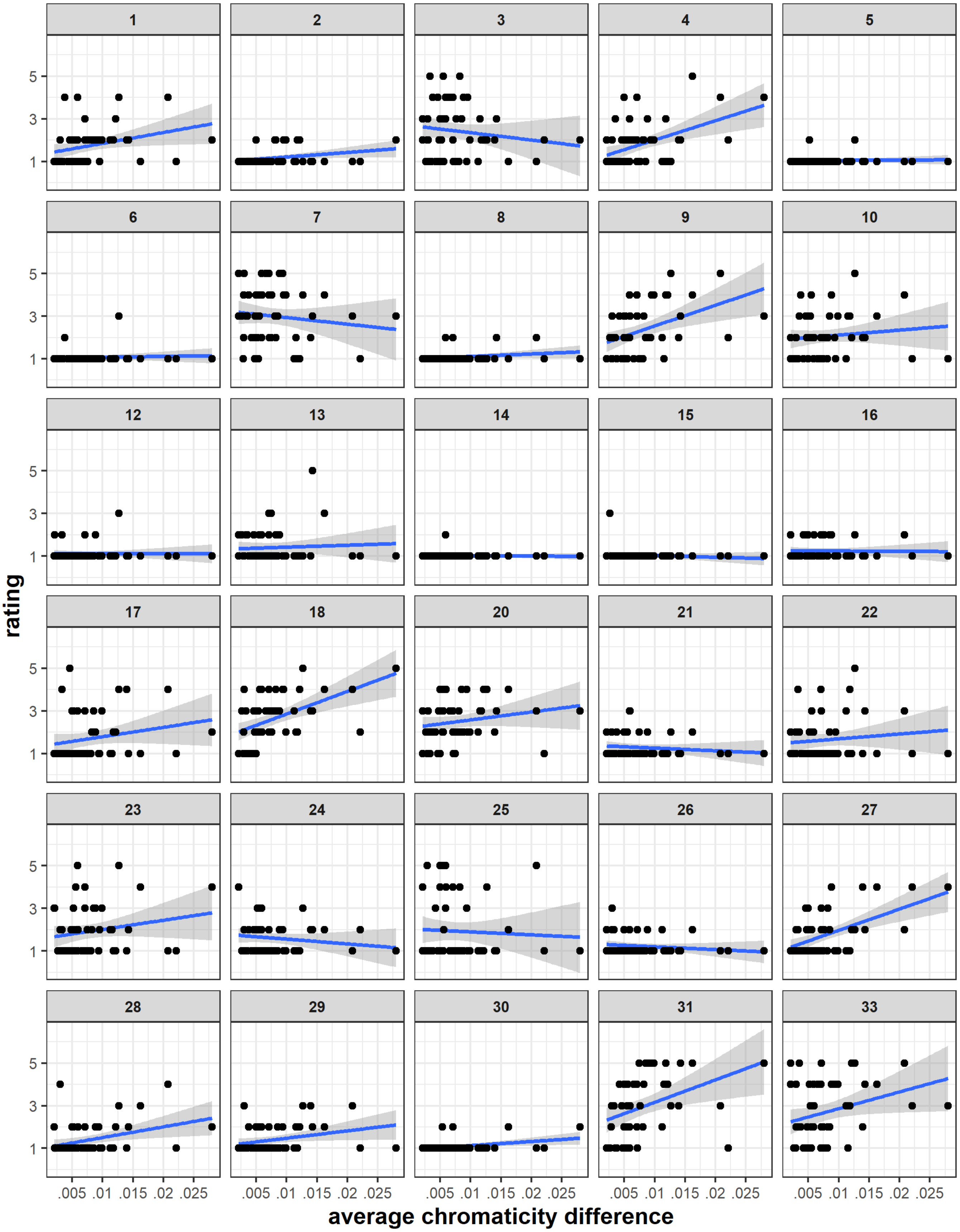

Figure S2, part1. Raw data for all the subjects included in the analysis of Experiment 2 (N = 58). Panel numbers indicate subject identity. Each panel shows the subject's ratings (between 1 and 5, in increasing discomfort) against the images (N = 50) average chromaticity difference. Blue line show best linear fit and shaded area shows 95% confidence intervals. (Figure continues in part 2.)

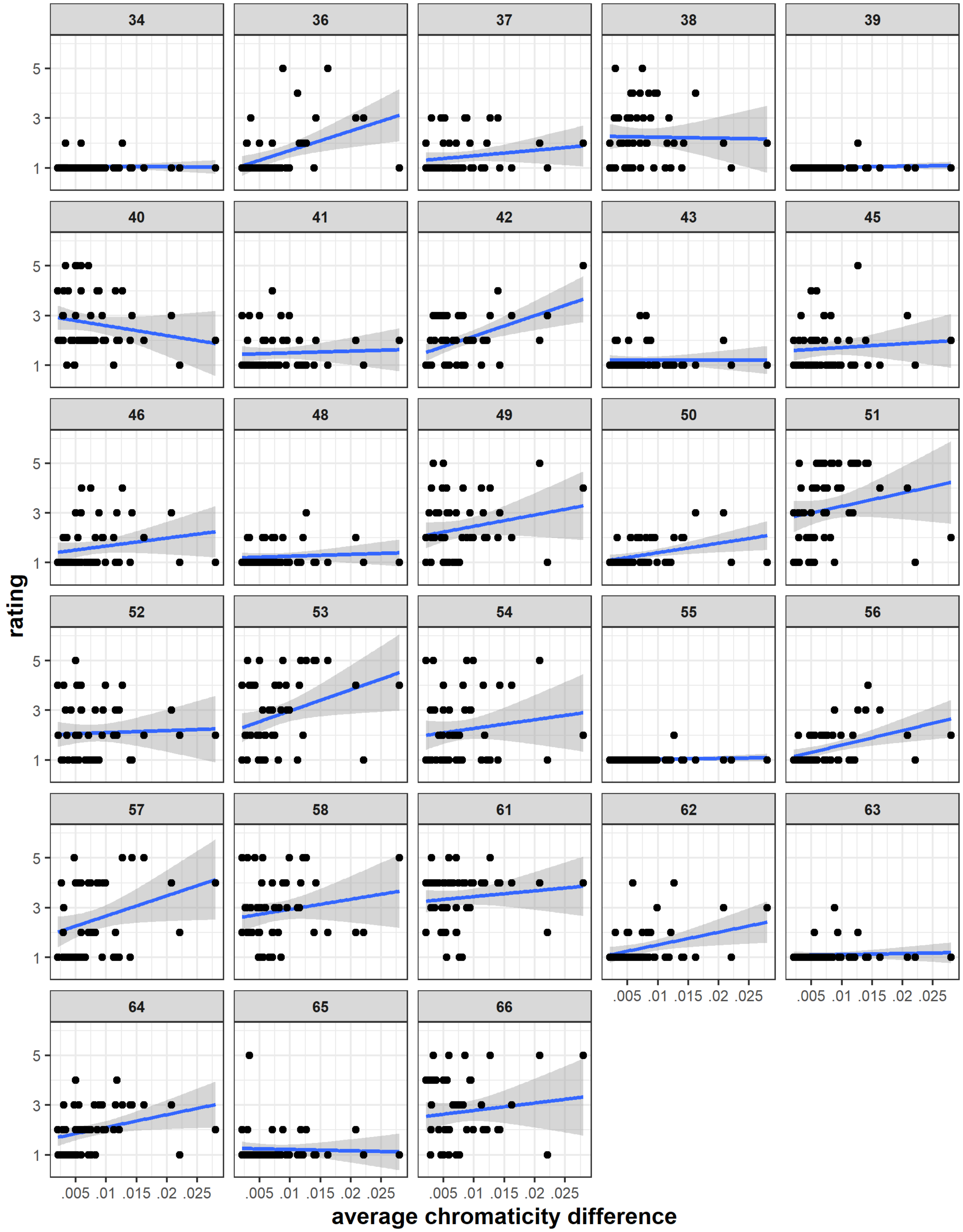

Figure S2, part2.

Modification of an image average chromaticity difference

To prepare the stimuli of Experiment 3 we recoloured the images in Set 1 using a set of transformations that modified their chromatic content, hence their average chromaticity difference, while preserving their luminance. We describe our algorithm below. Please note that the transformations did not allow us to transform a stimulus with a given average chromaticity difference into another stimulus with an arbitrary target average chromaticity difference. Instead, we used a great number of transformations (350, see below) to get a large set of transformed stimuli. In such a way we could increase the possibility of having a transformed stimulus with each of the three target levels of average chromaticity difference we had fixed for the experiment.

Algorithm

Each (sRGB) image in Set 1 was first sent to the CIE LUV space. We next kept the l coordinate aside and modified the chromatic coordinates of the image in the (u',v') plane to put the centre of the space (coordinates $(0.2105, 0.4737)$) to $(0,0)$ (by simply subtracting the coordinates of the centre to all the coordinates of the image in the (u',v') plane). We then applied all the possible combinations of rotations and pseudo-rotations defined below in the resulting space (accordingly, the (pseudo-)rotations were done around the centre of the (u',v')-plane). We did this in such a way that the relative distance from a point to the spectrum locus in the (u',v') plane was preserved. Finally, we shifted back to the (u',v') plane (by adding the coordinates of the centre of this space), took the l coordinates again and send the resulting (l,u',v') coordinates back to the sRGB space.

The rotations considered had angles $\theta_0 \ in \ [-\frac{\pi}{2}, -\frac{\pi}{4}, 0, \frac{\pi}{4}, \frac{\pi}{2}]$, giving 5 possible rotations. In polar coordinates, such a rotation sent a point P with angle θ to a point P' with angle $\theta + \theta_0$. The distance of P, d, was sent to the distance of P' $(d_{SL}(\theta + \theta_0)/d_{SL}(\theta)) * d$, where $d_{SL}(\theta)$ is the distance of the spectrum locus to the centre of the space in direction θ.

The pseudo-rotations considered also had angles in $[-\frac{\pi}{2}, -\frac{\pi}{4}, 0, \frac{\pi}{4}, \frac{\pi}{2}]$, but were not proper rotations in the sense that the rotation angle was not constant across all possible directions, but a continuous and monotonous (increasing) function that stretched or compressed some angular sectors. This stretching and compressing changed the chromatic distances of the image pixels. Formally, such a pseudo-rotation sent a point P with angle θ to a point P' with angle $h(\theta) + \theta_0$, where h was a smooth monotonic increasing function from $[0,2\pi]$ to $[0,2\pi]$ that preserved the whole interval (hence, with $h(0) = 0$ and $h(2\pi) = 2\pi$). The distance of P, d, was sent to $(d_{SL}(h(\theta) + \theta_0)/d_{SL}(\theta)) * d$. We considered two types of h functions, 'logistic' and 'power', and 7 parameters for each. This gave a total of 5 (rotations) x 5 (pseudo-rotations) x 2 (functions) x 7 (parameters) = 350 transformed images.

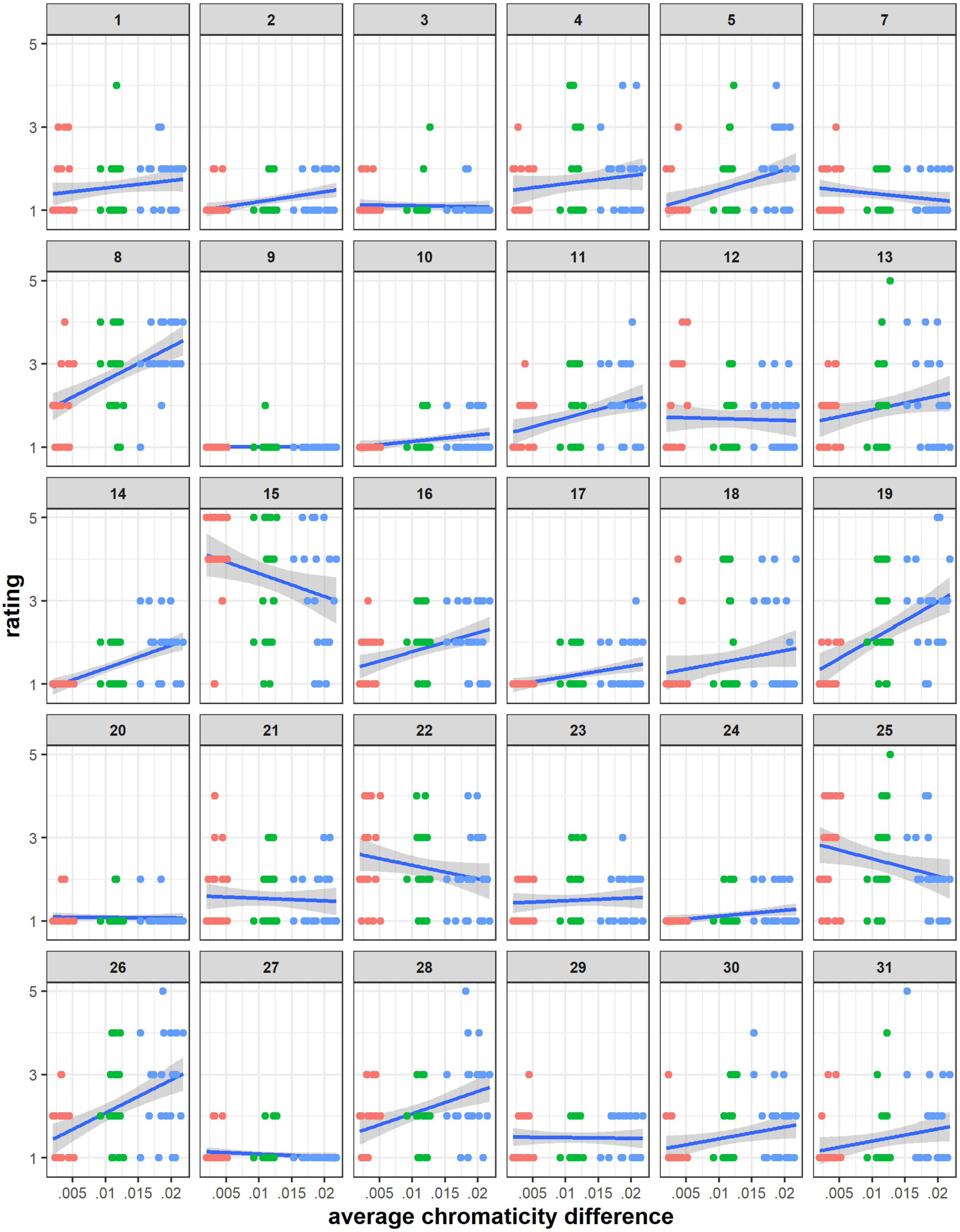

Figure S3, part 1. Raw data for all the subjects included in the analysis of Experiment 3 (N = 60). Panel numbers indicate subject identity. Each panel shows the subject's ratings (between 1 and 5, in increasing discomfort) against the images (N = 75) within the triples (N = 25) consisting of one image with a low level of average chromaticity difference (red colour, left), one with a medium (green, centre) and one with a high level (blue, right). Blue line show best linear fit and shaded area shows 95% confidence intervals. However, note that the analysis was made taking the group level (low, medium and high as predictor) and not the continuous level of the colour metric. (Figure continues in part 2.)

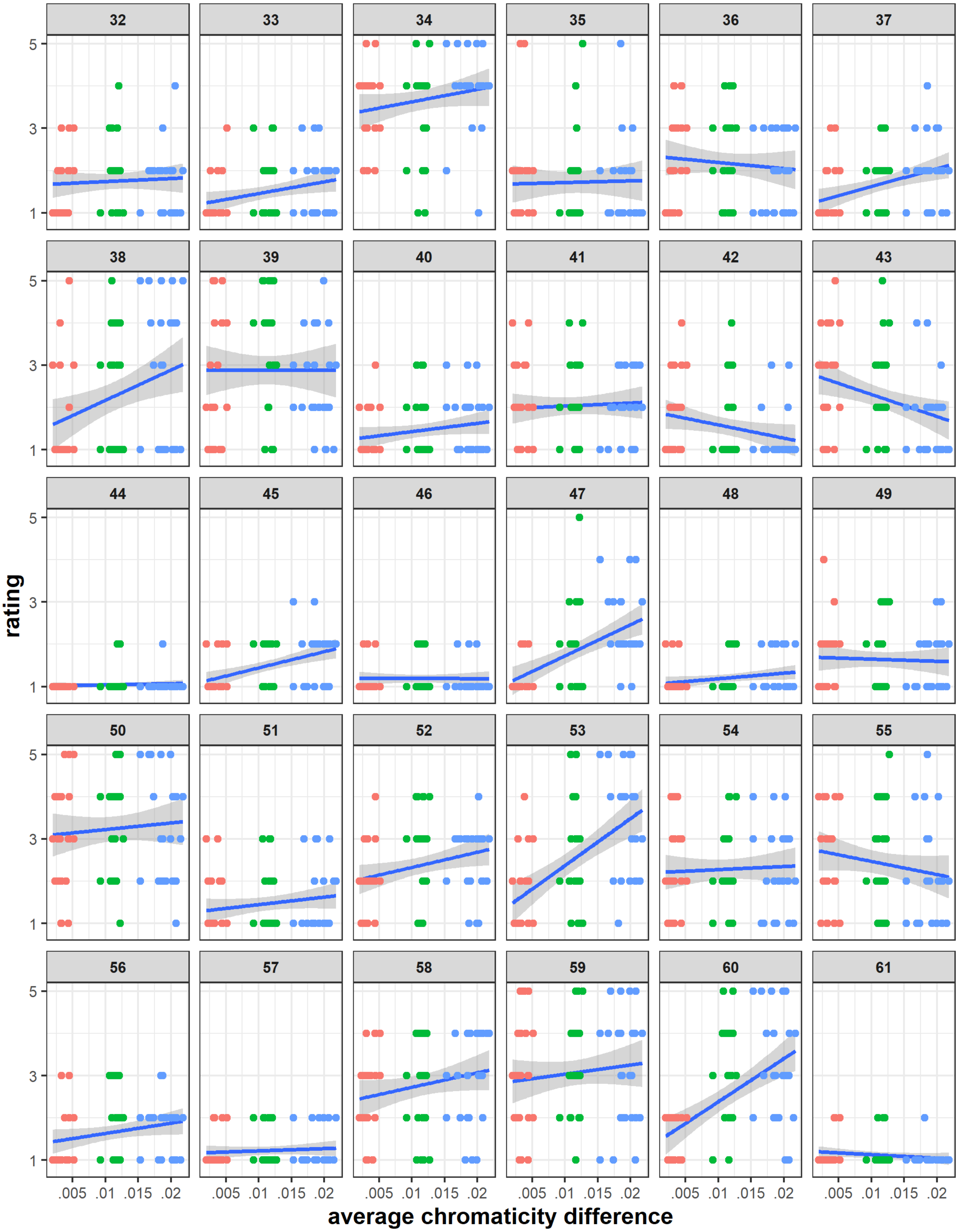

Figure S3, part 2.

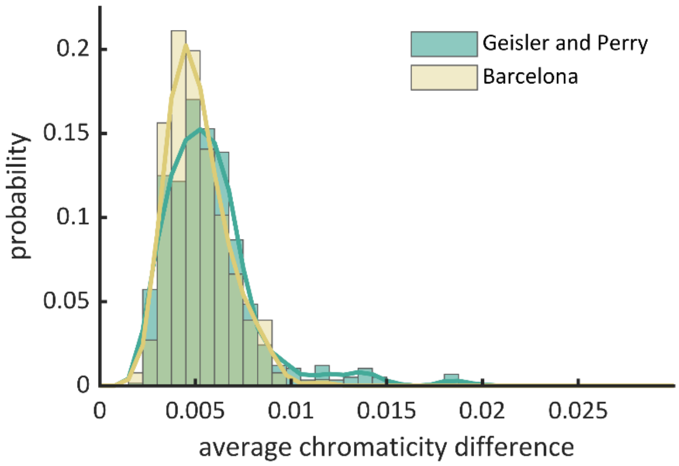

Figure S4. Comparison between the distributions of average chromaticity difference of Geisler and Perry database (N=576) and of Barcelona database (N=256). As the two distributions are quite similar (Jensen-Shannon divergence 0.0865), we used the two sets put together (N = 832) as our estimate for the distribution of average chromaticity difference in natural scenes.

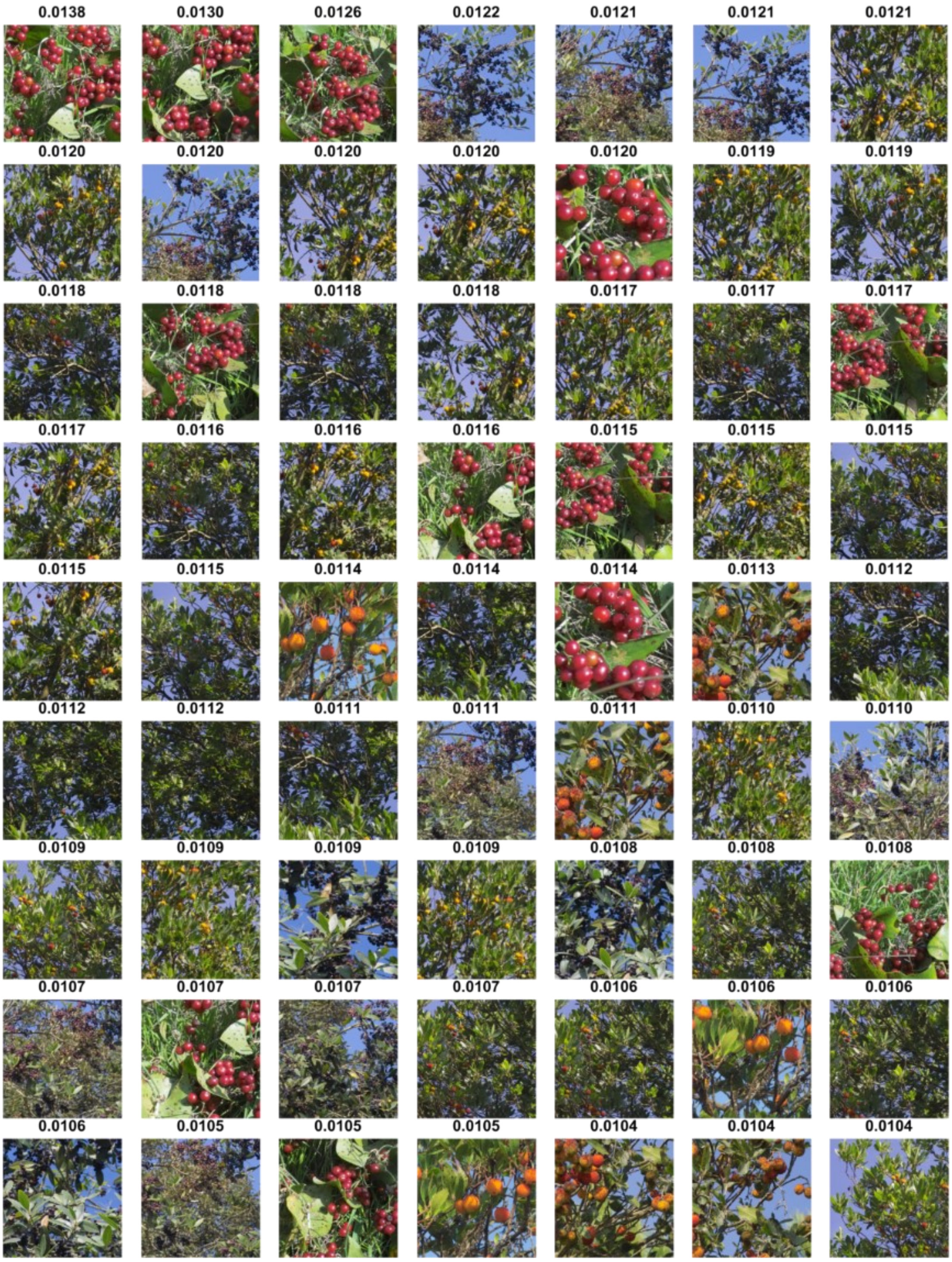

Figure S5. Random patches with highest average chromaticity difference in set 'Naturalistic 2' of the Barcelona calibrated database [ref] ordered, from let to right, top to bottom, in decreasing order. All the photographs in this set were acquired in May on the Mediterranean coastline, close to Barcelona, around location (41.525°N, 2.269°E). The average chromaticity difference of each patch is shown at the top. These excerpts of natural scenes from the Barcelona calibrated database show ripe or ripening fruits and foliage of plants commonly found around the Mediterranean, amongst which common smilax (*Smilax aspera*), from left to right, three first patches of the first row, privet (*Ligustrum vulgare*), patches 4 to 6, strawberry tree (*Arbutus unedo*), patch 7.

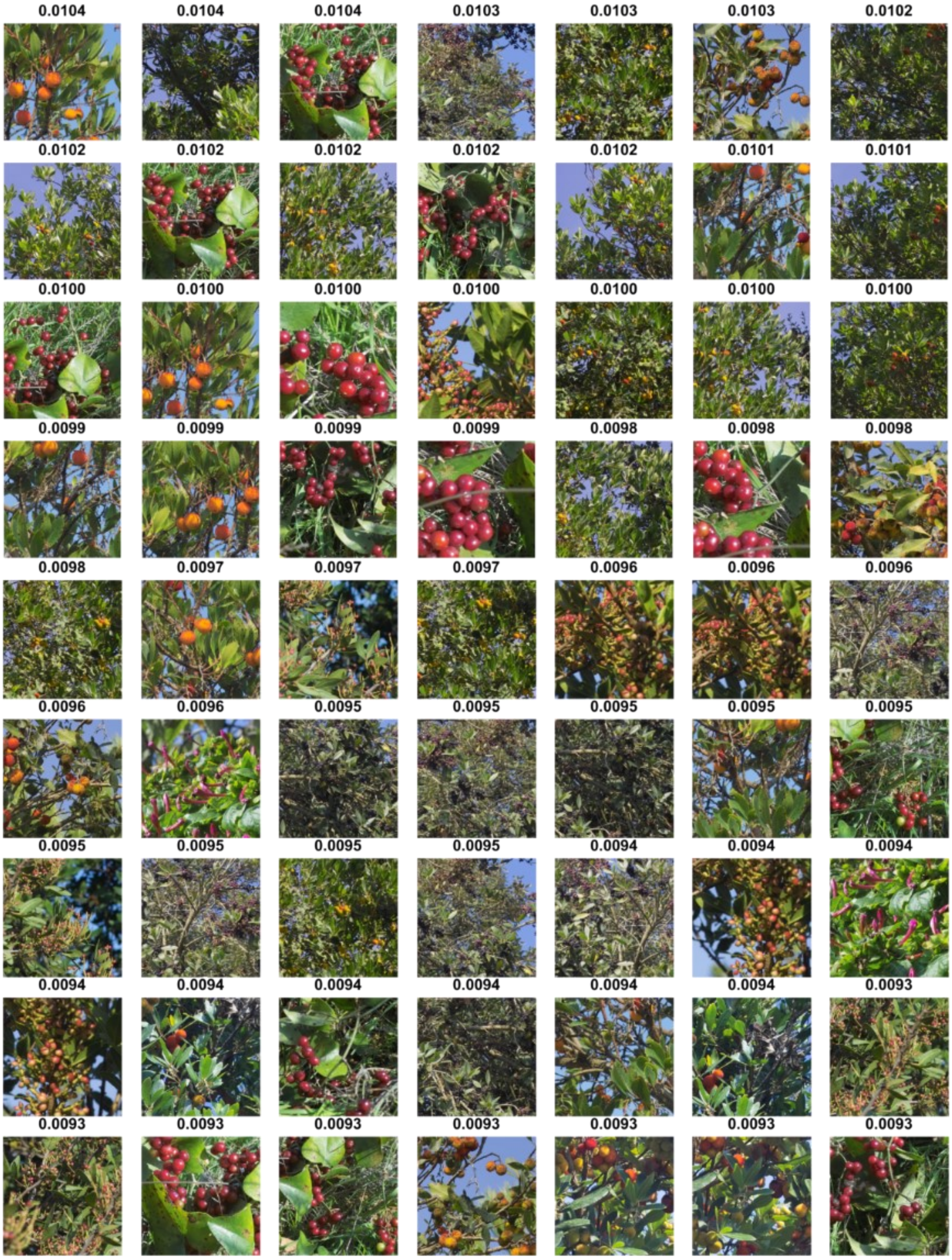

Figure S6. Continues from Fig.S5.

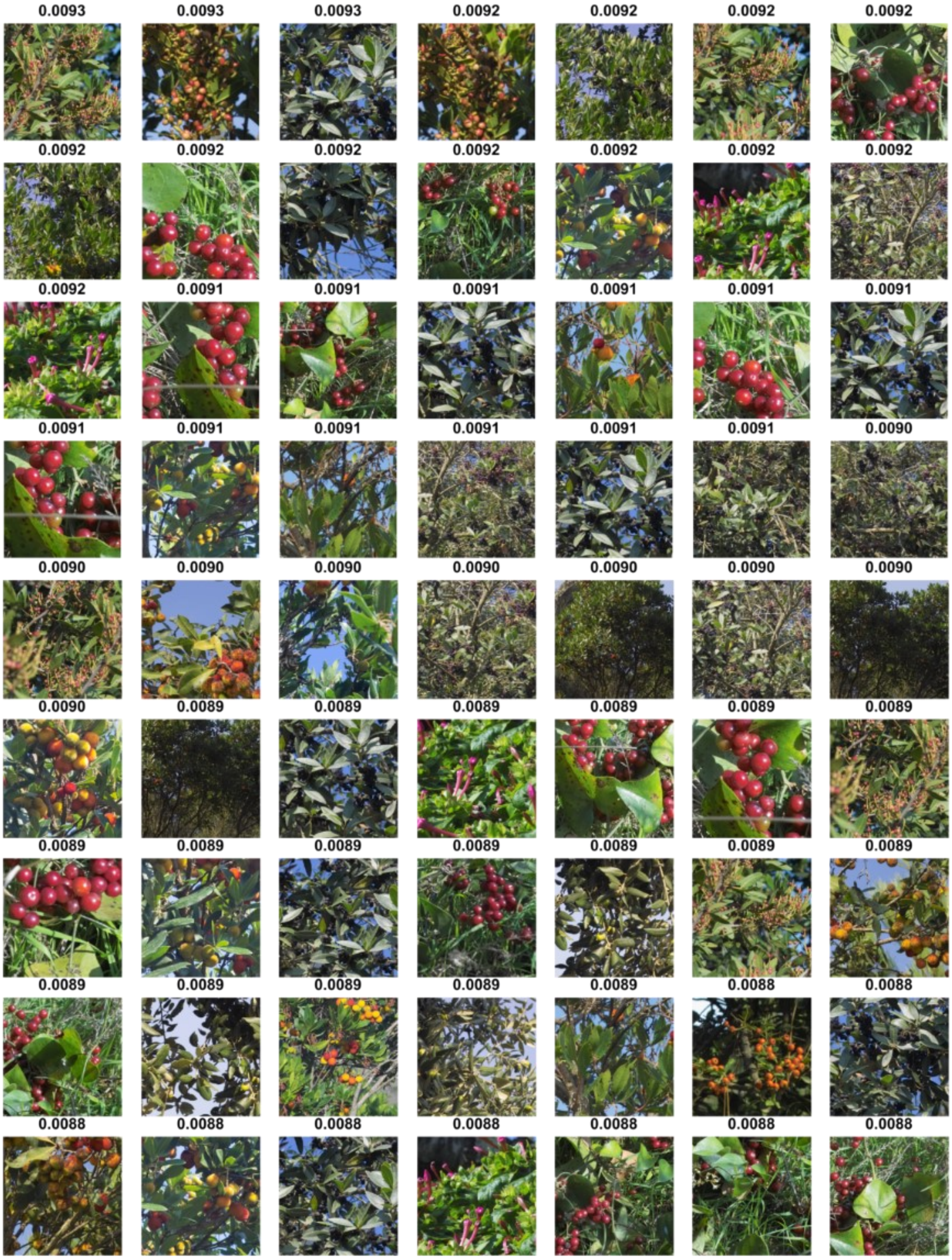

Figure S7. Continues from Fig.S5 and Fig.S6.

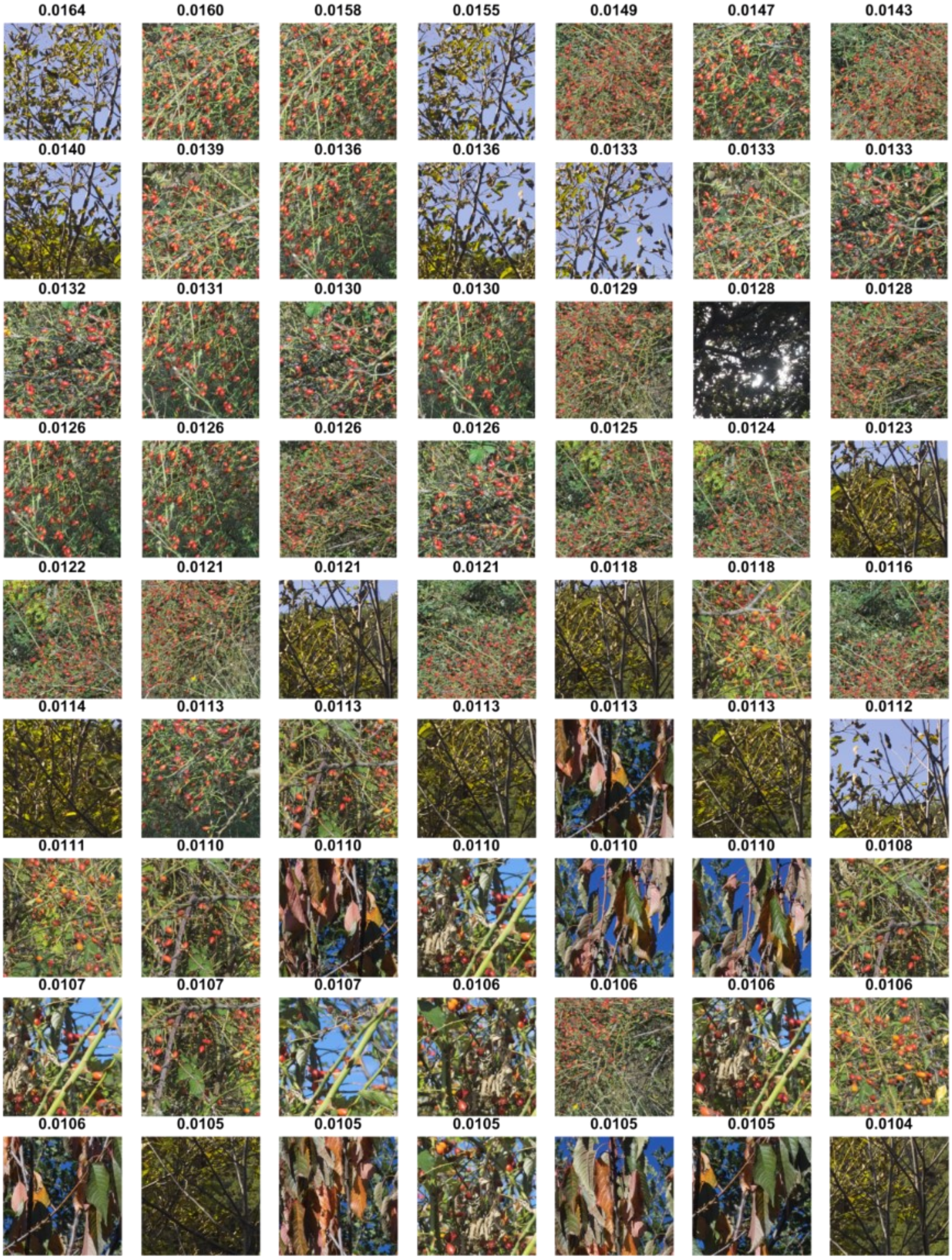

Figure S8. Random patches with highest average chromaticity difference in set ‘Naturalistic 3’ of the Barcelona calibrated database [ref] ordered, from let to right, top to bottom, in decreasing order. All the photographs in this set were acquired in October in the Mediterranean part of the Pyrenees, at the foothills of the Canigó mountain, around location (42.539°N, 2.334°E). The average chromaticity difference of each patch is shown at the top. These excerpts of natural scenes from the Barcelona calibrated database show ripe fruits of dog rose (*Rosa canina*), foliage of ash (*Fraxinus ornus*) against the sky and foliage of cheery tree (a species of *Prunus*) changing colour with autumn approaching.

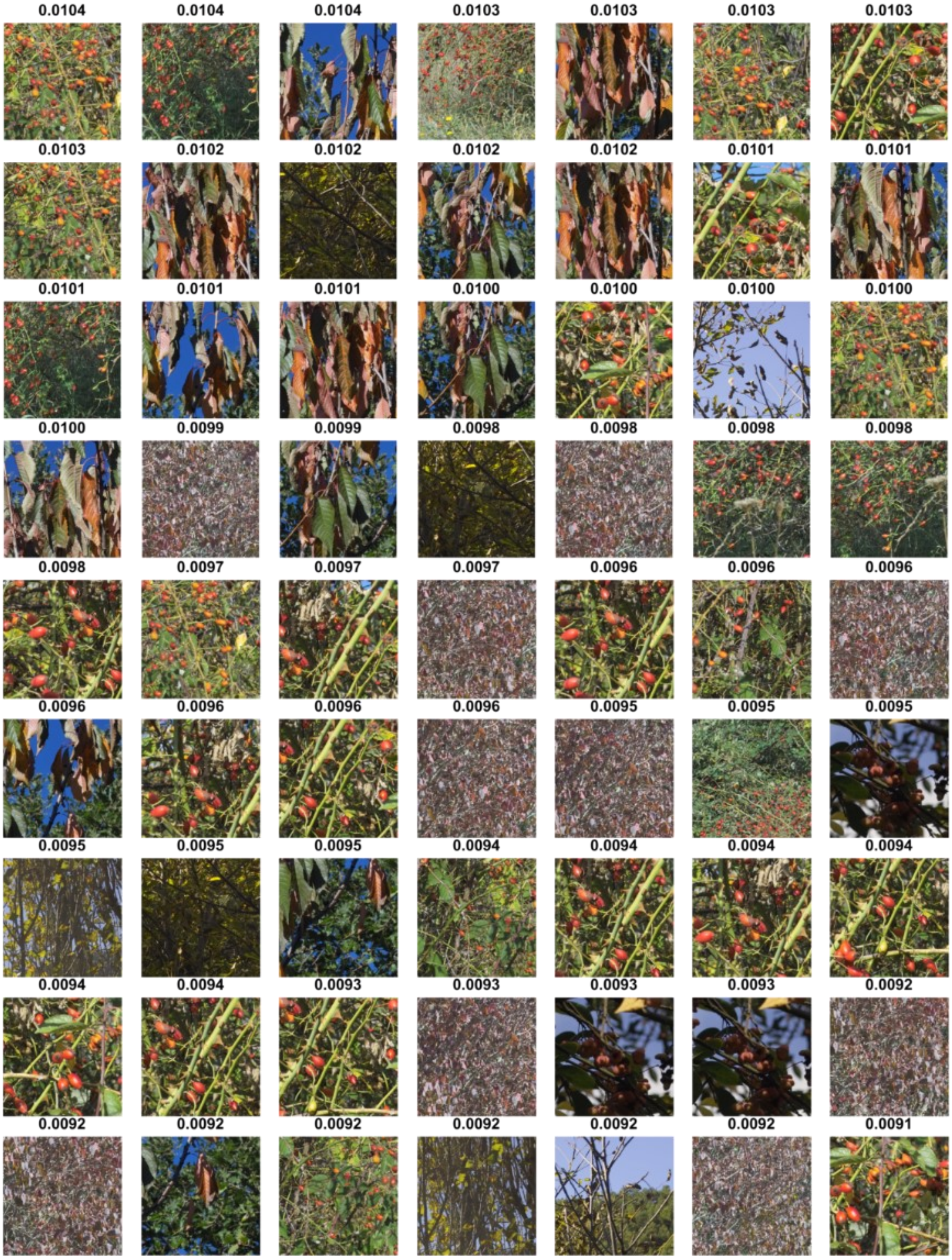

Figure S9. Continues from Fig.S8.

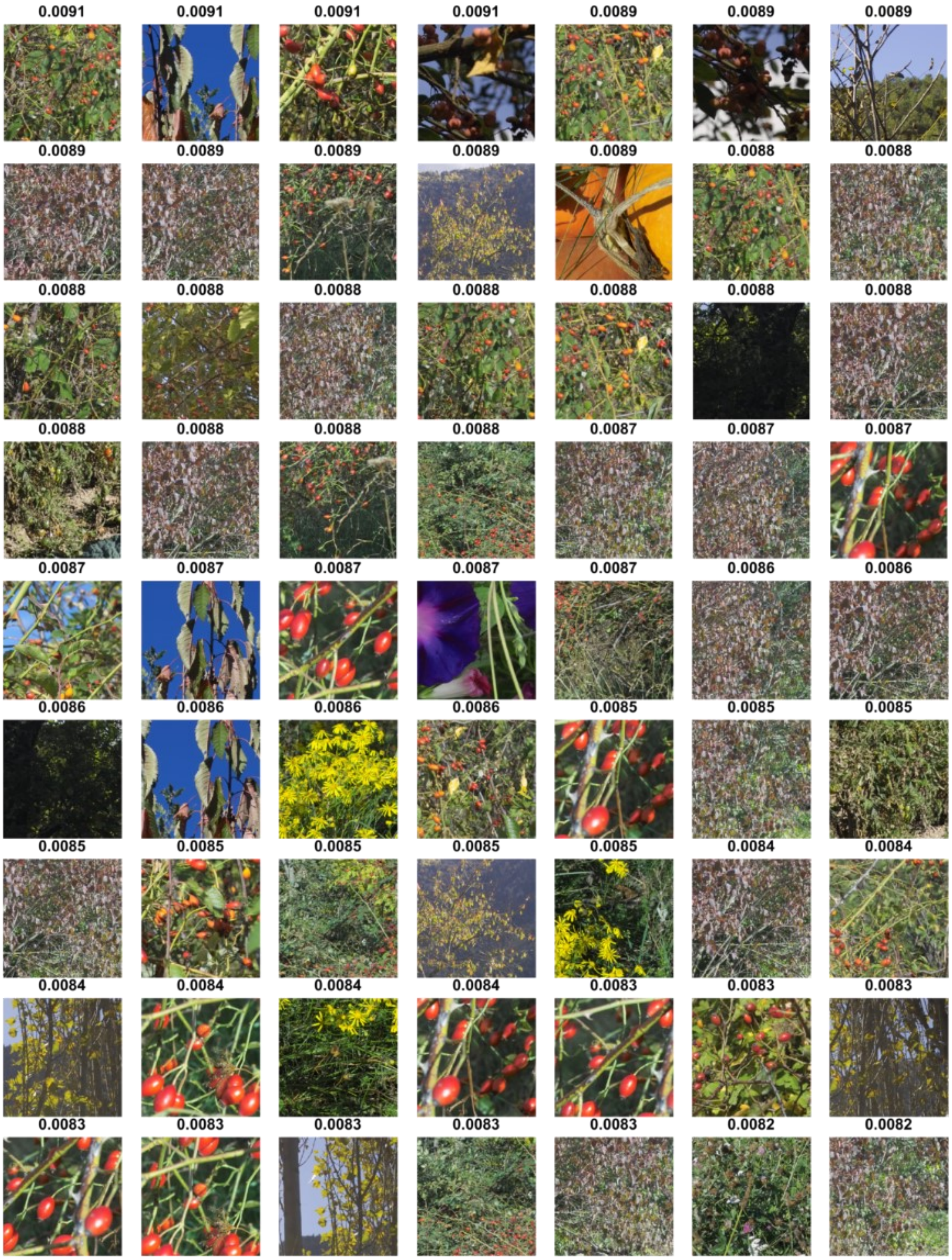

Figure S10. Continues from Fig.S8 and Fig.S9.

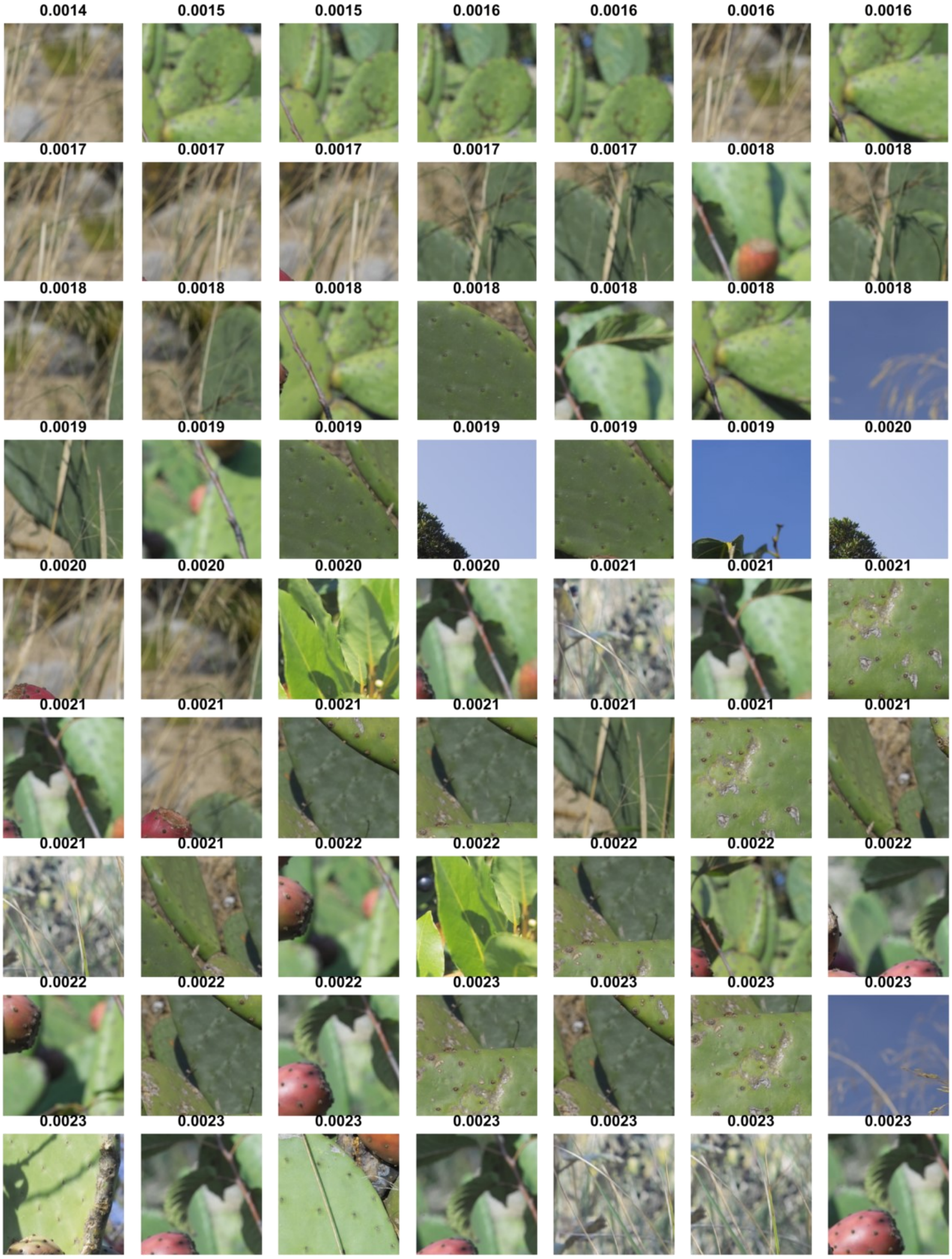

Figure S11. Random patches with lowest average chromaticity difference in set 'Naturalistic 2' of the Barcelona calibrated database [ref] ordered, from left to right, top to bottom, in increasing order. All the photographs in this set were acquired in May on the Mediterranean coastline, close to Barcelona, around location (41.525°N, 2.269°E). The average chromaticity difference of each patch is shown at the top. These excerpts of natural scenes from the Barcelona calibrated database show fairly chromatically uniform sky, dried wild-grasses, closed-ups of prickly pear (*Opuntia ficus-indica*), of bay laurel (Laurus nobilis), and persimmon tree (a species of *Diospyros*) with unripe fruit.

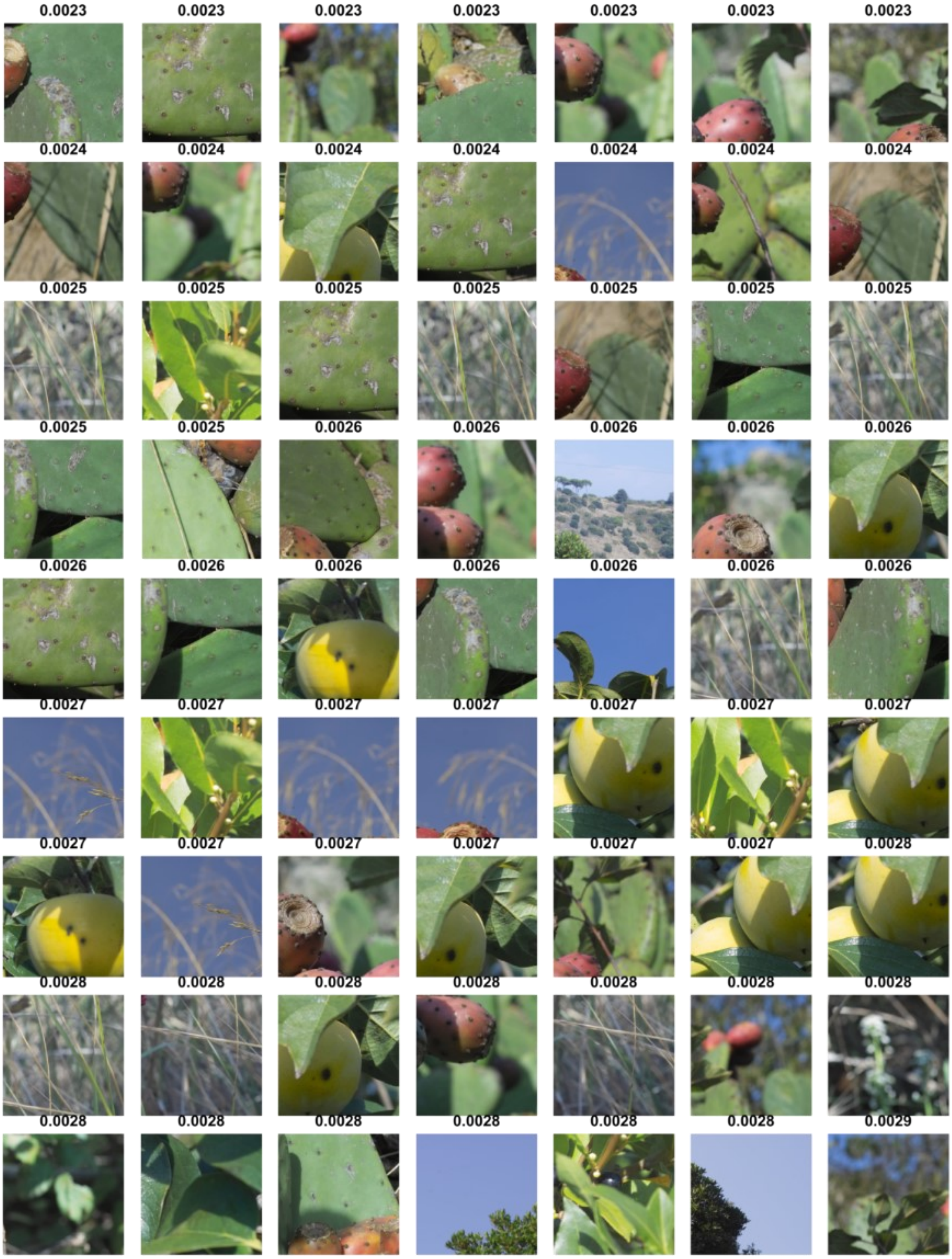

Figure S12. Continues from Fig.S11.

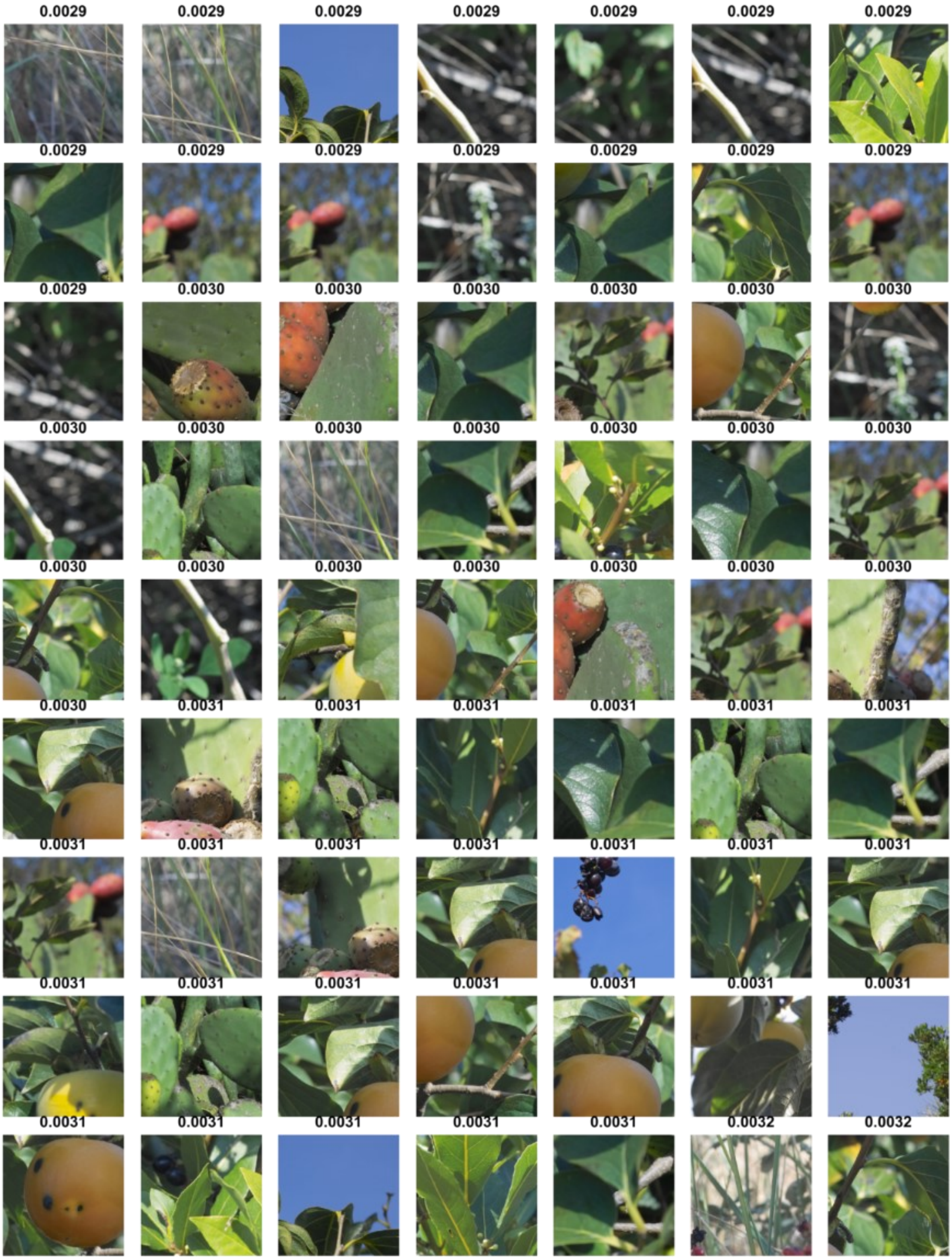

Figure S13. Continues from Fig.S11 and Fig.S12.

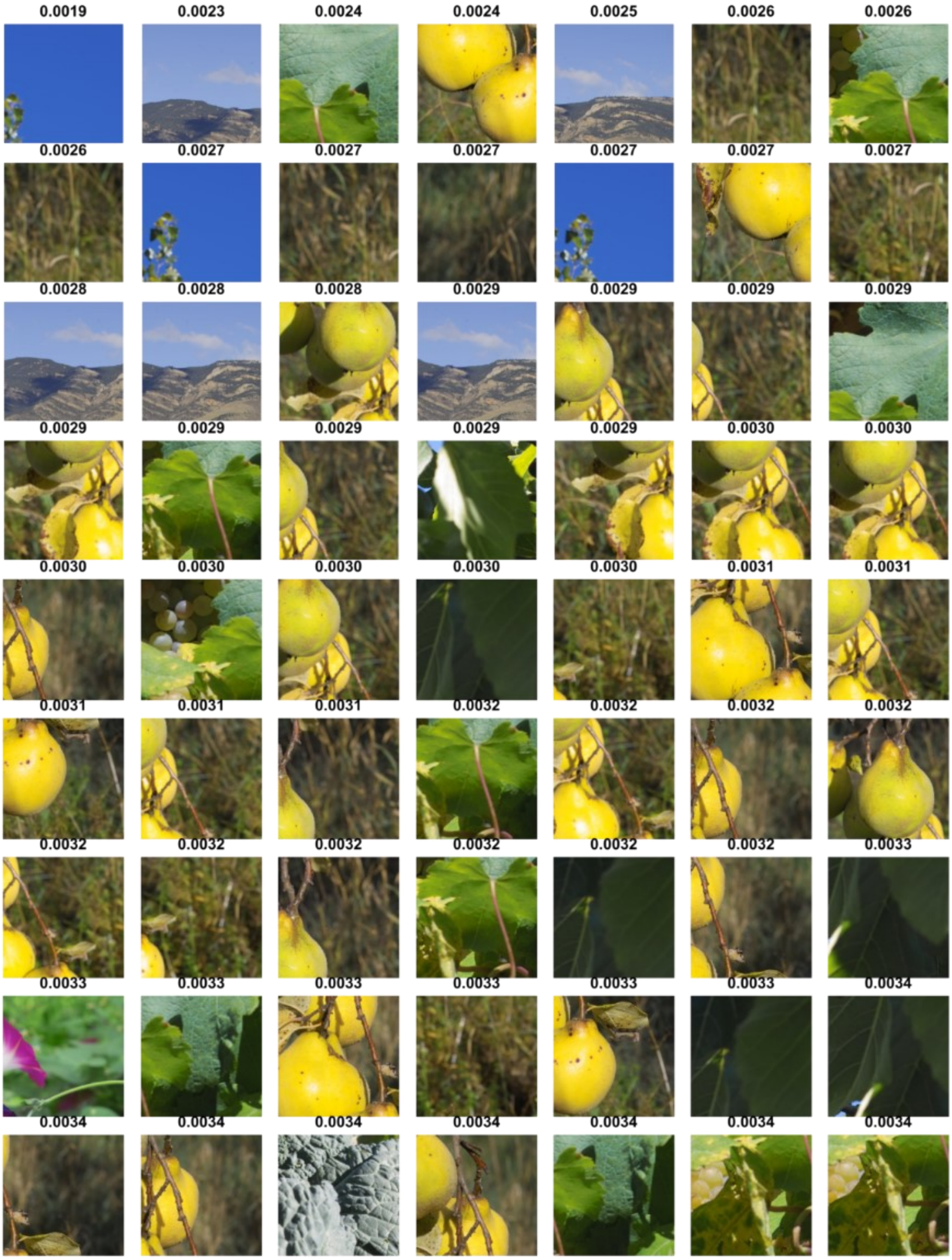

Figure S14. Random patches with lowest average chromaticity difference in set 'Naturalistic 3' of the Barcelona calibrated database [ref] ordered, from let to right, top to bottom, in increasing order. All the photographs in this set were acquired in October in the Mediterranean part of the Pyrenees, at the foothills of the Canigó mountain, around location (42.539°N, 2.334°E). The average chromaticity difference of each patch is shown at the top. These excerpts of natural scenes from the Barcelona calibrated database show fairly chromatically uniform sky, dried wild-grasses, close-ups of leaves of vineyard, fig tree (*Ficus carica*), close-ups of fruits of quince (*Cydonia oblonga*), mountain landscape and close-ups of vegetable garden.

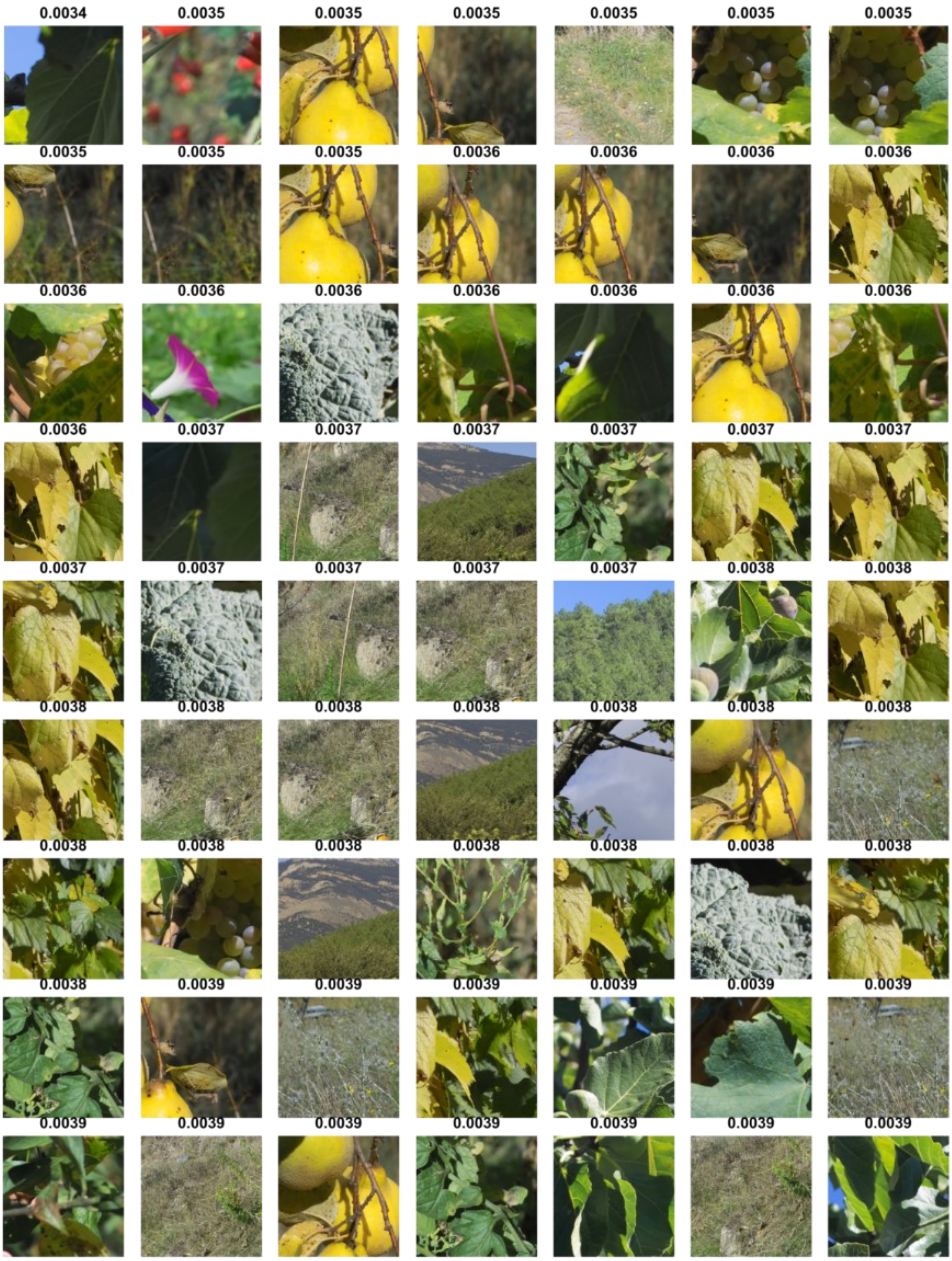

Figure S15. Continues from Fig.S14.

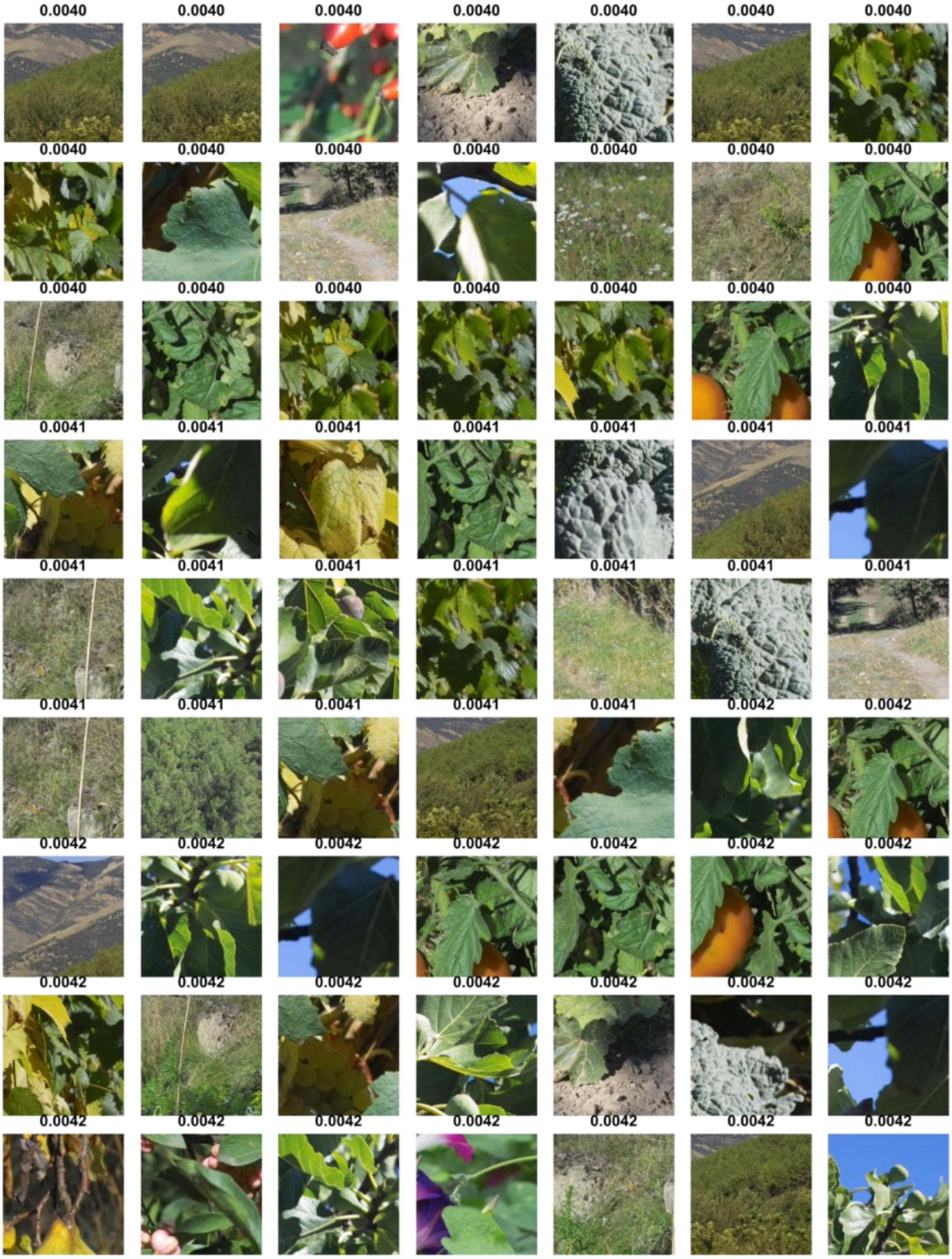

Figure S16. Continues from Fig.S14 and Fig.S15.
